# QuantGUV: Quantifying Encapsulation Efficiency of Small Molecules in Giant Unilamellar Vesicles

**DOI:** 10.1021/acsami.6c03651

**Published:** 2026-05-28

**Authors:** Zak Marshall, Reshma Bano, Pasha Dylan, Luisa Trifan, Callum Mckeaveney, André P. Gerber, Wooli Bae

**Affiliations:** † School of Mathematics and Physics, Faculty of Engineering and Physical Sciences, 3660 University of Surrey, Surrey GU2 7XH, U.K.; ‡ School of Biosciences, Faculty of Health and Medical Sciences, 3660 University of Surrey, Surrey GU2 7XH, U.K.

**Keywords:** giant unilamellar vesicles (GUVs), encapsulation efficiency, synthetic cells, image analysis, confocal microscopy, high-throughput, quantGUV

## Abstract

Synthetic cells, constructed through the self-assembly of small molecules, are designed to mimic life-like behaviors by encapsulating functional molecules. For such synthetic cells to accurately replicate cellular reactions, it is critical that the concentrations of encapsulated molecules mirror those in living systems, as reaction kinetics and cellular network states are highly sensitive to these concentrations. However, current methods for precisely determining encapsulation efficiency in synthetic cells at the single-cell resolution have been limited. To address this challenge, we present QuantGUV, a software-driven, image-based analysis method that determines the concentrations of fluorescent molecules encapsulated within giant unilamellar vesicles (GUVs). We use QuantGUV to measure the encapsulation efficiencies of three fluorescent molecules, sulforhodamine B, mEGFP, and polystyrene beads for GUVs formed via the water-in-oil emulsion transfer method. The encapsulation efficiencies for polystyrene beads were close to 100% in most of the conditions, while sulforhodamine B and mEGFP’s encapsulation efficiencies depended on the parameters during GUV formation, such as concentrations of lipids and oil–water ratio during GUV formation. By providing crucial insights into encapsulation efficiencies, QuantGUV offers a valuable tool to support the construction of quantitative synthetic cell systems with accurately controlled internal environments.

## Graphical abstract

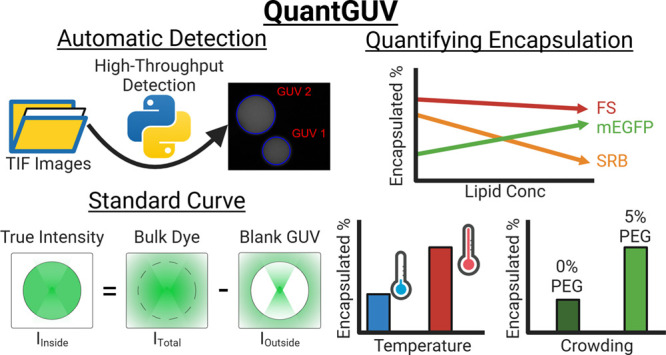

## 1 Introduction

A hallmark of living organisms is compartmentalization, a fundamental principle in which a membrane physically and functionally separates a living system from its surroundings.^1,2^ Fueled by recent advances in membrane biophysics, polymer physics, and bottom-up synthetic biology, it is now possible to construct artificial compartments with various physical properties such as size, curvature, and fluidity to mimic different characteristics of biological systems.^3–7^ Giant unilamellar vesicles (GUVs) are one of the key systems for studying compartmentalization, as they resemble the size of living cells.^8–16^ The GUVs can encapsulate functional molecules, including fluorescent compounds or a cell-free transcription-translation (TXTL) system to form synthetic cells that replicate core biological processes like information processing, membrane dynamics,^17–24^ molecular transport,^25–28^ and also develop possible drug delivery systems.^29–31^

To correctly mimic biochemical reactions, it is essential to encapsulate functional molecules at desired concentrations, as the rates and equilibrium dynamics depend on them. However, many of the currently available methods to form GUVs are subject to potential leakage and encapsulation concentration heterogeneities^32–38^ ­(Figure 1A). A limitation of these methods is their inability to guarantee the retention of initial molecular concentrations following GUV formation. Although it is straightforward to compare the relative encapsulation efficiency of different methods or compartments, this information is not sufficient to build predictable synthetic cells. Additionally, the overall yield of multistep enzymatic reactions, such as the in vitro synthesis of proteins, decreases exponentially with the number of steps involved.^39,40^

**Figure 1:**
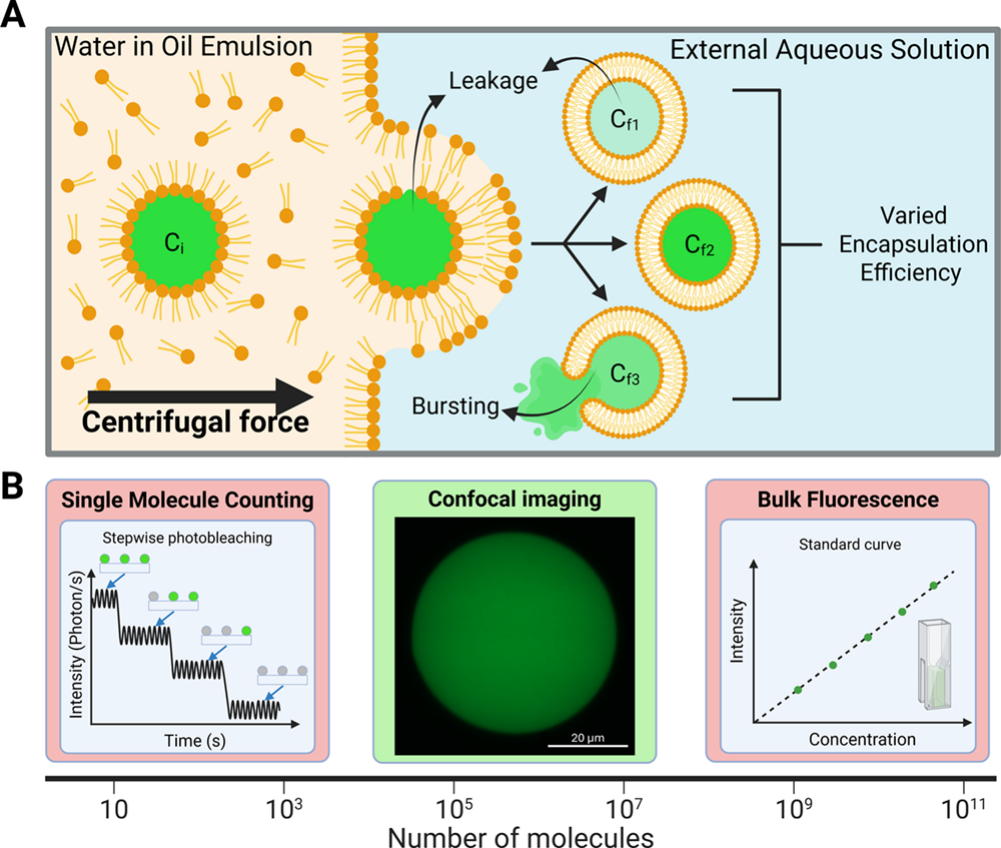
Challenges in encapsulation of molecules and determining the encapsulation efficiency (A) Schematic of GUV formation using inverted emulsion transfer method displaying that the initial concentration, *C*_*i*_, can change due to Leakage, *C*_*f*1_, random encapsulation, *C*_*f*2_ and bursting, *C*_*f*3_. (B) Methods for quantifying the concentration of fluorescent molecules, Left red, single molecule counting, Right red, bulk fluorescence using standard curves. Central green, confocal microscopy with reduced background. Created in BioRender. Marshall, Z. (2025) https://BioRender.com/qa7p0vv.

Therefore, there have been several attempts to quantify or enhance the encapsulation of functional molecules in GUVs. Sun et al. described a method of photolysis and single-molecule detection using confocal imaging to quantify the dyes encapsulated. Encapsulated fluorescent dyes were released by laser lysis of GUVs, and the measured fluorescence was fitted to a diffusion model to estimate encapsulation efficiency.^41^ They reported an encapsulation efficiency of 36.3 ± 18.9% for unilamellar/oligolamellar vesicles. Matosevic et al. used a microfluidic device to produce GUVs, and by comparing the measured fluorescence intensity of the droplets pre- and post-transfer, they were able to report an average encapsulation efficiency of 83%.^42^ Göpfrich et al. reported a method for producing GUVs using charged lipids in a “one-pot” method. This method was reported to retain high encapsulation efficiency; however, it was not quantified.^43^ Liu et al. used colorimetry with the Bradford method for quantifying protein encapsulation efficiency in the supernatant of GUVs formed from the emulsion transfer method^44^ and reported an average of 97.75 ± 1.73% for enzymes such as RNA Polymerase. Recently, Supramaniam et al. developed a microfluidic analysis chamber through which vesicles formed through inverted emulsion phase transfer were captured in an analysis chamber and lysed by both chemical and optical methods.^45^ By using single-molecule microscopy across a microarray, they counted spots and extrapolated to a standard curve to give absolute quantification with a mean encapsulation efficiency of 11.4%.

However, the examples above only give average values or involve specialized equipment and procedures with a relatively high barrier to entry, such as microfluidic techniques or single-molecule fluorescence microscopy. Current techniques are unsuitable for widespread adoption because they fail to address the intrinsic heterogeneity of the GUV size and content. The physical scale of the GUVs prevents the use of established methods such as single-molecule counting and obtaining a calibration curve from bulk solutions (Figure 1B). While established bulk characterization methods, such as HPLC or spectrometry, provide averages of encapsulation across thousands to millions of vesicles, they lack the resolution to capture variations across the population. This limitation is the target of QuantGUV to provide a method of quantification at the single vesicle level. Developing a standardized high-throughput approach to quantify encapsulation efficiency with a low barrier for entry of different sorts of molecules would enable wider adoption of GUVs for engineering and synthetic biological purposes, and enable more precise modeling of experimental results with mathematical modeling.

Herein, we present QuantGUV, a simple, standardized, software-driven pipeline for a high-throughput quantification of concentrations of encapsulated fluorescent molecules within GUVs (Figure 2). In brief, we generated standard curves of the intensities of fluorescent molecules at different concentrations within GUVs. Specifically, we took images of bulk dye solution and subtracted background intensities generated from images of empty GUVs in the bulk dye solutions. After images of the encapsulated molecules of interest within GUVs were acquired, an automated process determined the concentrations of the molecules by interpolating the estimated concentration from the standard curve. We produced GUVs using the water-in-oil emulsion transfer method, one of the most widely used GUV formation methods.^8,46^ Encapsulation efficiency was calculated for each individual GUV as the ratio of the encapsulant concentration within the GUV to the total encapsulant concentration initially added to the emulsion phase, expressed as a percentage. We used QuantGUV to characterize encapsulation efficiencies of three molecules with different sizes - sulforhodamine B (559 Da), monomeric enhanced green fluorescent protein (mEGFP) (27 kDa), and fluorescent spheres (2.6 MDa) at different conditions during the GUV formation.

**Figure 2:**
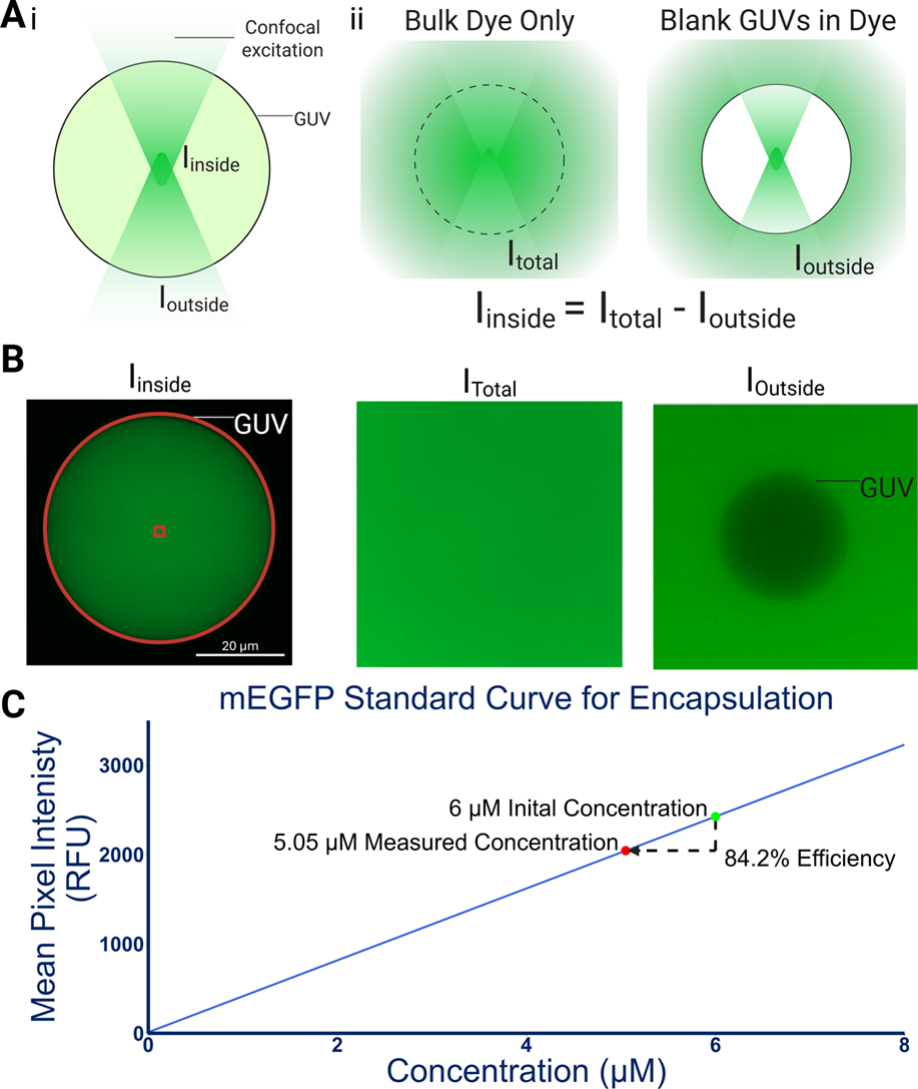
QuantGUV calibration curves (A) A schematic illustrating how the bright background correction works. To correctly estimate *I*_Inside_, *I*_Outside_ and *I*_Total_ are measured separately using empty GUVs in bulk solutions and bulk solutions only (B) Confocal images demonstrating the working principle. From left to right, a GUV encapsulating mEGFP (*I*_Inside_), a bulk mEGFP solution (*I*_Total_) and a blank GUV within a solution containing mEGFP (*I*_Outside_). The image contrast and highlights have been enhanced for ease of viewing. (C) mEGFP standard curve correlating known protein concentration with mean pixel intensity (RFU) calculated from *I*_Total_ – *I*_Outside_. The equation of the line is used to set the RFU for the initial concentration of protein in the solution (green dot) the same equation is used to interpolate the *I*_Inside_ of the GUV above and determine the internal concentration (red dot). Encapsulation efficiency is determined by $\frac{\mathrm{Measured}\;\mathrm{Concentration}}{\mathrm{Initial}\;\mathrm{Concentration}}\times 100$ . Created in BioRender. Marshall, Z. (2025) https://BioRender.com/953ins9.

## 2 Results

### 2.1 QuantGUV Standard Curve Generation

Accurate quantification of encapsulated molecules within GUVs presents a significant technical challenge. For a standard 10 μm GUV at a concentration of 1 μM, the internal volume contains upward of 300,000 molecules, a density that precludes individual molecule counting techniques.^47^ While confocal microscopy provides a localized fluorescence signal, this intensity cannot be directly converted to a concentration due to its dependence on instrumental parameters, including laser intensity and the numerical aperture of the objective lens as well as environmental impacts on the fluorophore’s quantum yield. To overcome these variables, a robust standard curve is required to relate internal fluorescence (*I*_Inside_) to known concentrations. However, establishing this curve using GUVs with defined internal concentrations is impractical, as the nonideal encapsulation efficiencies and inherent heterogeneity of GUV formation are the very variables requiring measurement. Furthermore, using a simple bulk solution as a standard is insufficient because confocal measurements include a nonzero contribution from out-of-focus fluorescence. We define this relationship by eq 1:

$$I_{\mathrm{Inside}}=I_{\mathrm{Total}}-I_{\mathrm{Outside}}$$ 1

where *I*_Total_ represents the signal from a bulk solution, and *I*_Outside_ denotes the background signal originating from outside the focal volume. While *I*_Inside_ cannot be measured directly for a calibration standard, we hypothesized that *I*_Outside_ could be isolated by imaging “blank” GUVs containing no fluorophores, suspended in a known concentration of fluorescent molecules (Figure 2A). In practice, we generated the standard curve by experimentally determining *I*_Total_ and *I*_Outside_ across a broad dilution series. *I*_Total_ was established by measuring the mean pixel intensity of the fluorescent species prepared in an Internal Aqueous Solution (IAS) using a confocal plate reader under the same chemical and physical conditions used for all GUV experiments present here. To determine the corresponding *I*_Outside_ component, empty GUVs were introduced into the same solutions. The QuantGUV software was then utilized to identify fluorescence voids within the bright bulk solution and measure from the lumen of these empty vesicles. Because these GUVs lack internal fluorophores, any detected signal within the lumen represents the *I*_Outside_ background. By subtracting these *I*_Outside_ values from the *I*_Total_ measurements, we isolated the theoretical *I*_Inside_ values, allowing for the construction of a linear standard curve (Figure 2C) that accounts for out-of-focus light and instrumental bias.

The linearity of the QuantGUV calibration was rigorously validated across a multipoint concentration series for each molecule. Linear regression analysis (Supporting Information Figure S5 and Table S6) yielded high coefficients of determination (*R* ^2^ > 0.97 for SRB and mEGFP; *R* ^2^ > 0.95 for FluoSpheres), confirming a proportional relationship between internal fluorescence intensity and molar concentration within the tested ranges.

### 2.2 QuantGUV Quantification of GUVs

As the accurate measurement of the *I*_Total_, *I*_Inside_, and *I*_Outside_ is important in obtaining a good calibration curve, we have developed the QuantGUV pipeline to correct for artifacts introduced during the imaging process. First, to minimize the effect of the lipid membranes, which could affect the quantum yield of fluorescence molecules, QuantGUV determines the geometric center of the detected GUVs and measures the fluorescence signal from that region. Second, GUVs are immobilized on a surface for stable signal acquisition. Additionally, a series of computational algorithms is applied to exclude any out-of-focus vesicles and correct for background fluorescence (See Section 3 for details). After the quantification of images, the intensity values were normalized by dividing the intensity by the integration time to match the standard curve. This allows for wider dynamic range and higher signal-to-noise value for improved data quality. After determining its concentration, it is compared to the initial concentration of molecules in the IAS. The vesicle detected in Figure 2B, left, was normalized and plotted on the standard curve in Figure 2C, where the concentration detected and the initial concentration can be compared. In the initial emulsion, 6 μM of mEGFP was used, and by using the standard curve, the estimated concentration within the vesicle was 5.05 μM, which is an 84.2% encapsulation efficiency.

### 2.3 Quantifying Encapsulation of Biomimetic Size Probes in GUVs

To represent typical biochemical molecules involved in synthetic cell reactions, we measured the encapsulation efficiencies of three molecules with varying hydrodynamic diameters. Sulforhodamine B (SRB, 1 nm), monomeric enhanced green fluorescent protein (mEGFP, 4 nm), and FluoSpheres (FS, 20 nm).

SRB is a small fluorescent dye with a size similar to that of nucleotides or short peptides. mEGFP is a fluorescent polypeptide with a size similar to that of small enzymes or protein subunits. FS are styrene spheres modified with a fluorophore, with a size similar to that of a bacterial ribosome and other large supramolecular complexes.

To evaluate the impact of size on encapsulation efficiency, we tested three molecules across three Lipid-in-Oil (LiO) concentrations (0.5, 2, and 5 mg/mL) while using a fixed internal aqueous solution (IAS) percentage in the emulsion phase (5%). The resultant GUVs were imaged via confocal microscopy using standardized gain conditions (Figure 3A), and then images were processed by QuantGUV using exclusion criteria circularity 0.7 and convexity 0.9. The interpolated encapsulation efficiencies were averaged and plotted as bars (Figure 3B). In total, 12,432 individual vesicles were analyzed across all conditions in this study (median of 156 per condition, range 52–1653), aggregated from 3–5 independent biological replicates.

**Figure 3:**
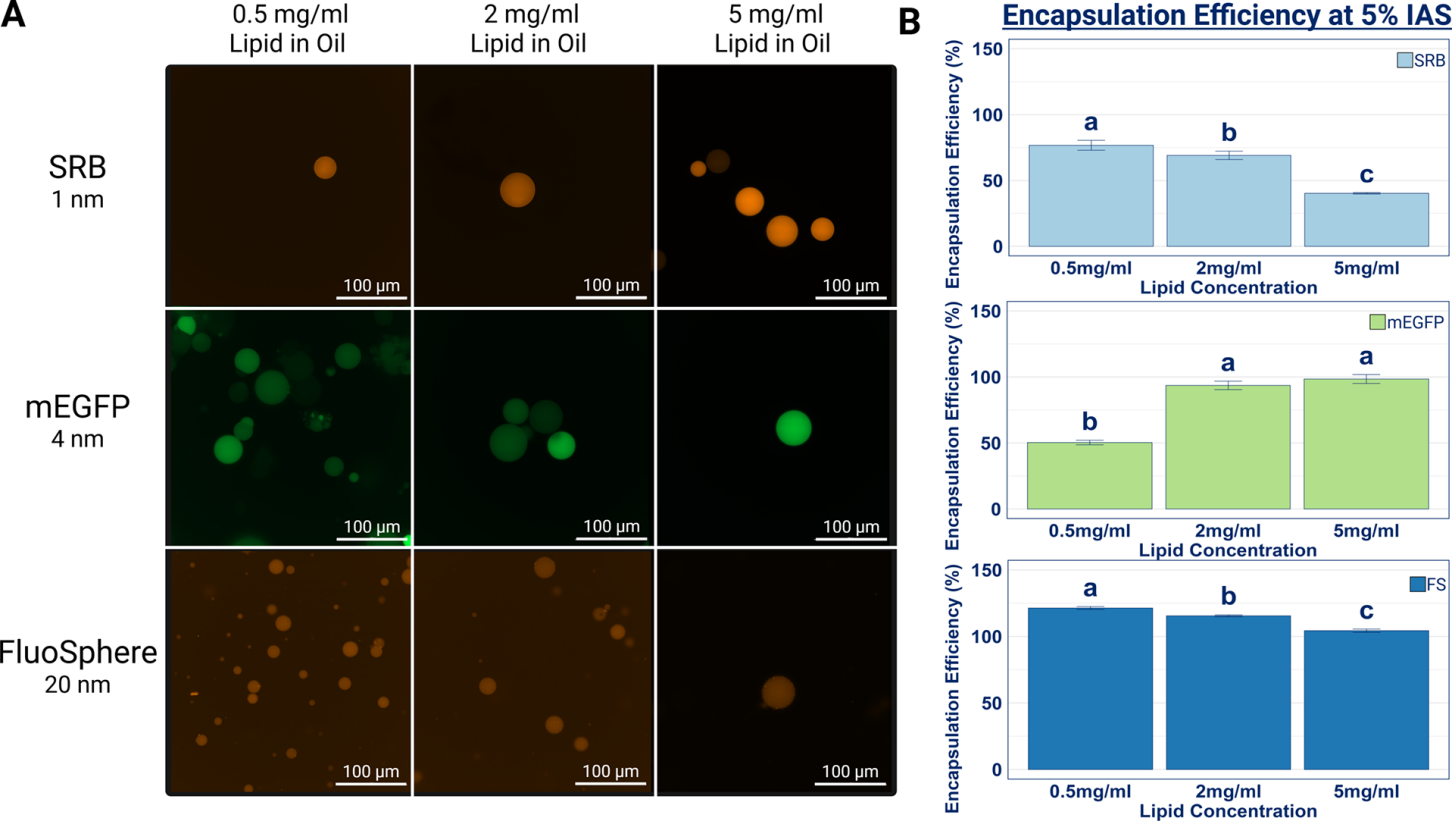
Encapsulation efficiency of fluorescent molecules of differing sizes (A) Representative fluorescence microscopy images of GUVs formed with varying lipid-in-oil concentrations (0.5, 2, and 5 mg/mL). Three molecules with different sizes -Sulforhodamine B (SRB, 1 nm), monomeric enhanced green fluorescent protein (mEGFP (4 nm), and FluoSpheres (FS, 20 nm) are encapsulated. (B) Quantification of encapsulation efficiencies with QuantGUV. The efficiency for SRB and FS significantly decreased with increasing lipid concentration, while the encapsulation efficiency for mEGFP increased. Data are presented as mean ± standard error of the mean from 3 to 5 biological replicates. Letters (a–c) denote statistically significant differences between groups as determined by a one-way ANOVA with Tukey’s posthoc test (*p* < 0.05) (see Supplementary Table S13 for full *F*-statistics and posthoc *p*-values). Columns not sharing a letter are statistically significant to each other.

The largest cargo, FluoSpheres, yielded the highest overall encapsulation values, ranging from 103.2 to 119.9% (Figure 3B, bottom). While a slight decrease in efficiency was observed as LiO concentration increased, the values remained consistently above 100%, indicating a concentration effect within the vesicle lumen from the initial seed concentration. In contrast, mEGFP displayed a strong positive correlation with lipid concentration, as encapsulation efficiency significantly increased from 43.7% at 0.5 mg/mL LiO to 88.4% at 5 mg/mL LiO. Conversely, the smallest molecule, SRB, followed an inverse trend, with the highest mean encapsulation of 74.2% occurring at the lowest lipid concentration (0.5 mg/mL) and decreasing to 38% at 5 mg/mL LiO. Collectively, these results demonstrate that the lipid density in the oil phase has opposing effects on encapsulation efficiency depending on the size of the fluorescent cargo.

### 2.4 Effect of IAS Ratio and Lipid Concentration on Encapsulation Efficiency

To further explore the parameters governing encapsulation, we investigated the interplay between the IAS ratio (1, 5, and 10%) and LiO concentration. Across all tested molecules, the LiO concentration emerged as the primary determinant of encapsulation efficiency, while the IAS ratio exerted a more subtle, molecule-specific influence (Figure 4). Representative confocal images for all tested IAS and LiO combinations are provided in Supporting Information Figure S9. Furthermore, for comparative clarity, the data for the 5% IAS ratio previously presented in Section 2.3 have been replotted in Figure 4 alongside the results for the 1 and 10% conditions. For the smallest molecule, SRB (1 nm), encapsulation was highly sensitive to both experimental variables. While the inverse relationship with LiO concentration remained consistent across all aqueous ratios, increasing the IAS ratio from 1 to 10% significantly boosted efficiency at the lowest lipid density (0.5 mg/mL), reaching a peak of 124.5% (Figure 4A). This value suggests a pronounced concentration effect under conditions of high IAS volume and low lipid availability. In contrast, the encapsulation of mEGFP (4 nm) showed a sustained positive correlation with lipid density regardless of the IAS ratio. For this midsize protein, the highest efficiencies were consistently achieved at 5 mg/mL LiO, with the IAS ratio having a negligible impact on the overall trend (Figure 4B). Finally, the FluoSpheres (20 nm) maintained the most stable encapsulation profile across varied conditions. Although they followed the inverse LiO trend seen with SRB, their efficiency remained consistently high across all of the IAS ratios (Figure 4C). This stability suggests that the entrapment of larger species is less susceptible to variations in the initial aqueous-to-lipid volume ratio, as their physical size likely precludes the transient leakage pathways that affect smaller molecules during the GUV formation process.

**Figure 4:**
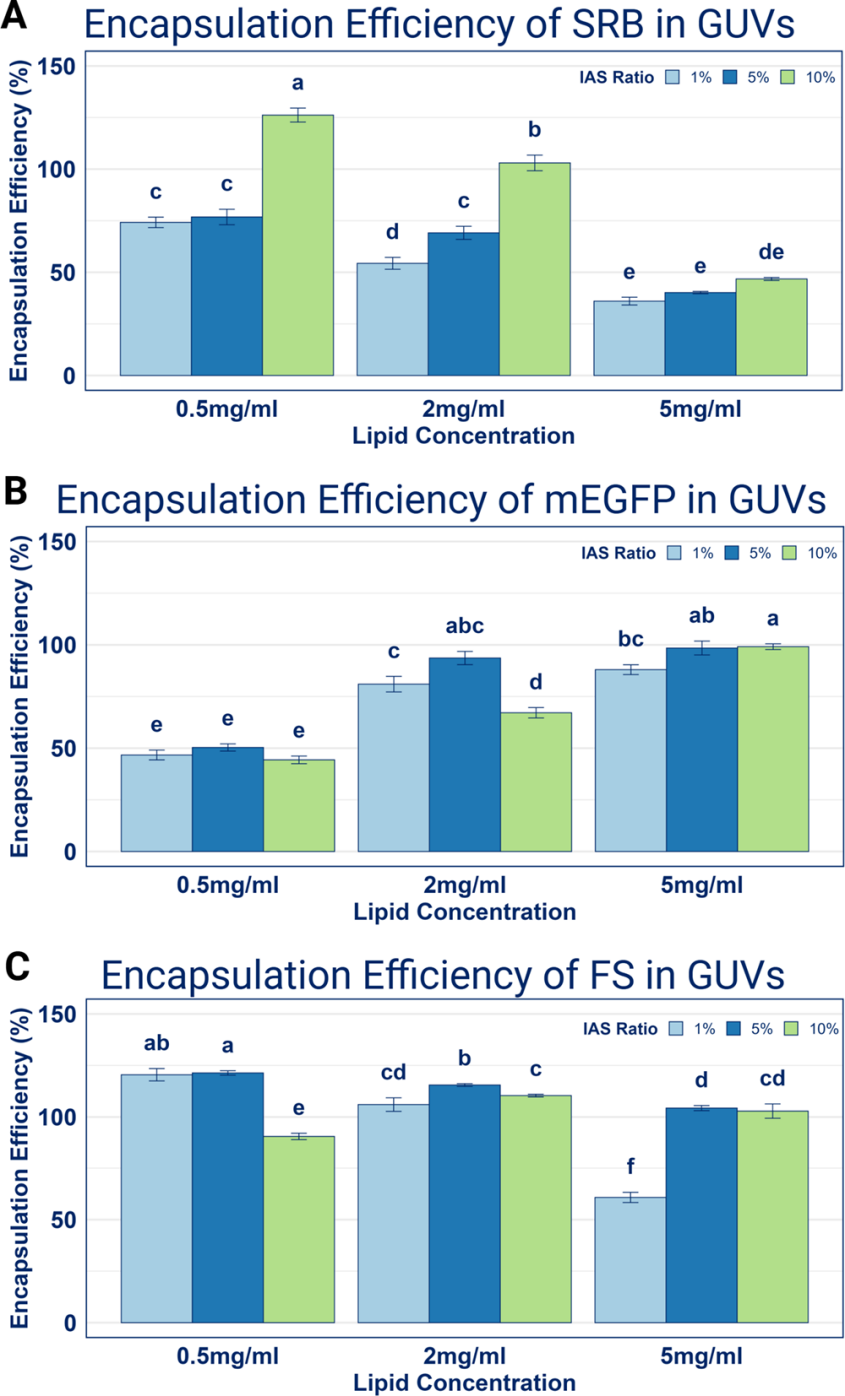
Encapsulation Efficiencies of varying IAS ratio in varied LiO concentrations. The encapsulation efficiency of three different fluorescent molecules was assessed: (A) Sulforhodamine B (SRB), (B) monomeric Enhanced Green Fluorescent Protein (mEGFP), and (C) FluoSpheres (FS). GUVs were prepared using varying total lipid concentrations (0.5, 2, and 5 mg/mL) and different Inner Aqueous Solution (IAS) to lipid solution volume ratios (1, 5, and 10%). Bars represent the mean encapsulation efficiency (%) from three to five independent experiments (*n* = 3 – 5), with error bars indicating ± standard error of the mean. Letters (a–f) denote statistically significant differences between groups, as determined by a two-way ANOVA followed by Tukey’s posthoc test was used for statistical analysis. Bars not sharing a common letter denote a statistically significant difference between the groups (*p* < 0.05)­(See Supplementary Table S14 for full *F*-statistics and posthoc *p*-values). Note: The data representing the 5% IAS condition (center bars) is reproduced from Figure 3 to facilitate direct comparison across varying IAS ratios.

### 2.5 Effect of Temperature and Molecular Crowding on Encapsulation

Molecular crowding is known to enhance biochemical reactions. To test its effect, we added different concentrations of polyethylene glycol (PEG) 8000 to the IAS to determine its impact on the concentration of mEGFP encapsulated (Figure 5A). Two mg/mL LiO with an IAS ratio of 2.5% was used at four PEG concentrations. Hence, GUVs were produced in PEG-free and 3, 4, and 5% w/v PEG conditions (Figure 5A). To correct for any fluorescence change caused by the addition of PEG to the system,^48^ bulk fluorescence of IAS containing 6 μM mEGFP in the presence of PEG was measured at varying PEG concentrations. QuantGUV analysis of the vesicles shows a significant increase in mEGFP encapsulation efficiency as PEG increases (Figure 5B). For instance, an increase in the encapsulation efficiency was observed, from 58.73% at 0% PEG to 107.27% at 5% PEG. This demonstrates a positive effect of crowding agents on the encapsulation efficiency of mEGFP. We also tested the effect of maintaining low temperature during the GUV formation on the encapsulation efficiency during GUV formation. This is to maintain the activity of biochemical reactions during the encapsulation process. Two temperatures, 4 and 20 °C, were chosen to represent the shift from room temperature to a reduced temperature (Figure 5C). To ensure the fluorescence signal was not affected by the temperature during formation, images were taken at room temperature. QuantGUV analysis of the vesicles shows that the mean encapsulation efficiency decreased from 52.6% at 20 °C to 40.8% at 4 °C (Figure 5C,D). While statistically significant, this decrease may be considered an acceptable compromise to preserve the functional integrity of encapsulated systems.

**Figure 5:**
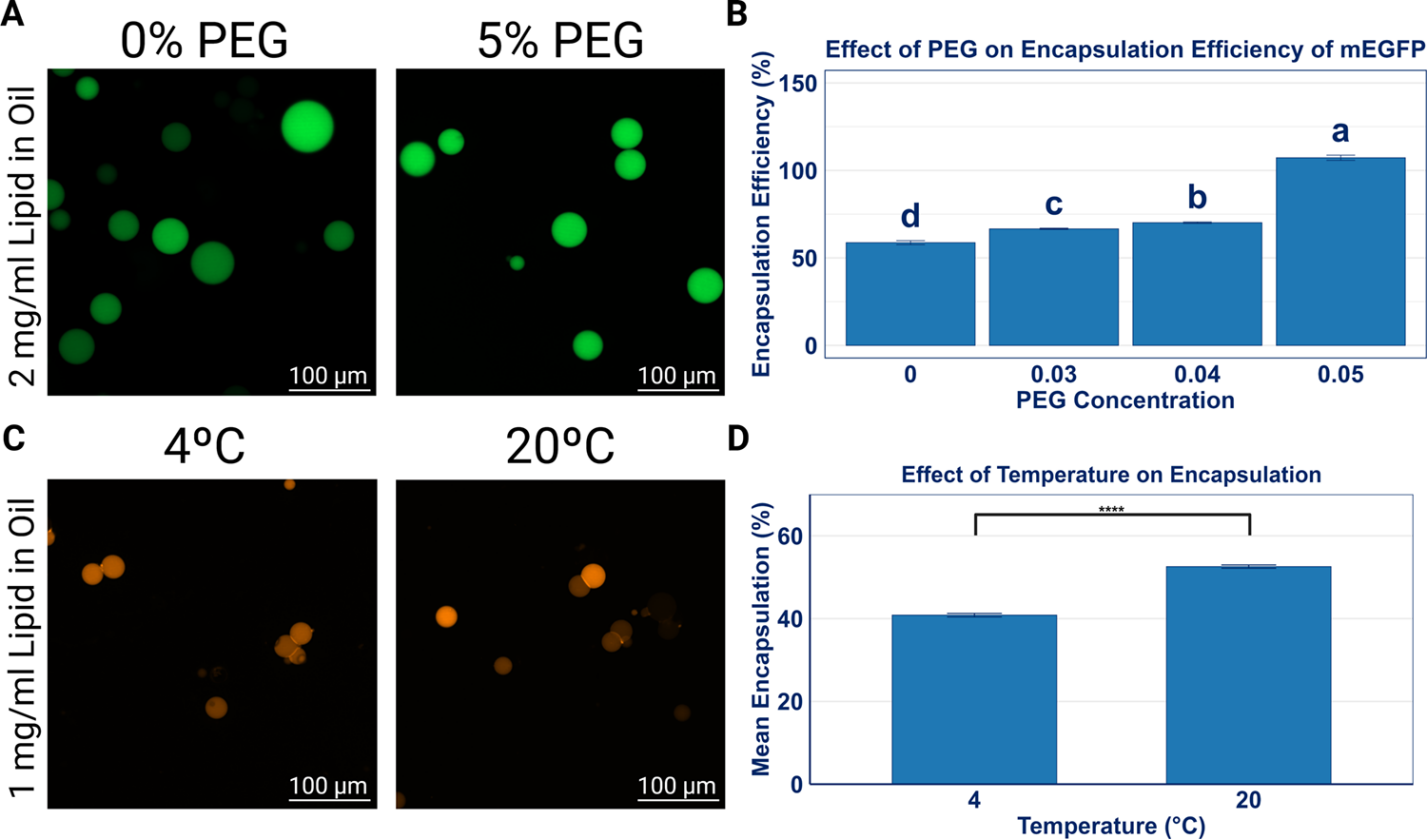
Effect of Temperature and PEG on GUV Encapsulation Efficiency. The encapsulation efficiency of GUVs was explored further (A) Confocal images of GUVs containing in the presence of 0% PEG, left, and 5% PEG, right, Scale bars = 100 μm (B) Mean encapsulation efficiency of mEGFP encapsulated within GUVs using a variation of PEG concentrations 0, 3, 4, and 5% w/v using a 2 mg/mL LiO and an IAS ratio of 2.5%. Data presented as mean ± standard error of the mean from 3 to 5 biological repeats. Letters (a–c) denote statistically significant differences between groups as determined by a one-way ANOVA with a Tukey’s posthoc test (*p* < 0.05) (See Supplementary Table S15 for full F-statistics and posthoc *p*-values) (C) Sulforhodamine B (SRB), was encapsulated within GUVs using a formation temperature of 4, left, and 20 °C, right, using a 1 mg/mL LiO and an IAS ratio of 2.5%. (D) Mean Encapsulation of SRB presented as mean ± standard error of the mean from 3 to 5 biological repeats, a two tail independent *t* test was used to analyze significance, **** indicates *p* < 0.0001. (See Supplementary Table S16 for full statistics and *p*-values).

## 3 Methods

### 3.1 Lipid in Oil Preparation

1,2-dioleoyl-*sn*-glycero-3-phosphoethanolamine-*N*-(biotinyl) (sodium salt) (Cat#870282) (18:1 Biotinyl DOPE) and 1,2-dioleoyl-*sn*-glycero-3-phosphocholine (Cat#850375) (18:1 DOPC) were purchased from Avanti Polar Lipids. Lipid dye 1,1′-Dioctadecyl-3,3,3′,3′-Tetramethylindodicarbocyanine, 4-Chlorobenzenesulfonate Salt (Cat#D7757) (DiD) was purchased from Thermo Fisher Scientific. Stock solutions in chloroform and ethanol were stored at −20 °C. Lipid in Oil (LiO) solutions were prepared by mixing 18:1 DOPC lipids (25 mg/mL), with 18:1 Biotinylated-DOPE lipids (1 mg/mL) and DiD (1 mg/mL) in 20 mL WHEATON liquid scintillation vials (Cat#DWK986546) from Sigma-Aldrich to a ratio of 99.8:0.1:0.1% w/v. The solvent was desiccated in a fume hood under a dry stream of nitrogen while rotating to form a thin dry lipid film. The lipid film was then dispersed in 2 mL BioReagent mineral oil (Cat#M5904) obtained from Sigma-Aldrich to achieve a final concentration of 0.5, 2, or 5 mg/mL. Glass vials were sealed with Parafilm, then LiO solutions were sonicated in a sonic bath at 40°*C* for 30 min and were used the same day in experiments.

### 3.2 Inverted Emulsion Phase Transfer

The internal aqueous solution (IAS) containing 1x Phosphate Buffered Saline (PBS, Cat#10010023, Thermo Fisher Scientific) and 200 mM sucrose (Cat#A15583.36, Thermo Fisher Scientific) was added to the LiO solutions; the amount of IAS added in a ratio to the oil forms the IAS ratio. IAS ratios of 1, 2, or 5% were used. Typically, 300 μL of LiO solution was used with a corresponding amount of IAS. The mix was then mechanically emulsified on a standard Eppendorf rack at approximately 1 pass per second for 10 passes.

The amount of IAS added to the oil phase during the emulsion step does not induce a change in molecule concentration as the water and oil phases are immiscible.

The emulsion was then incubated at 4 °C for 2 min. The external aqueous solution (EAS), 100 μL containing 1× PBS and 200 mM glucose (Cat#15023021, Thermo Fisher Scientific) was placed in a 1.5 mL Eppendorf tube, and 150 μL of the emulsion was layered on top of the EAS. The stack was then centrifuged at 7000 *g* for 8 min. The transferred GUVs form a pellet, which is collected by aspiration and redissolved in fresh EAS. For experiments using fluorescent dyes, the IAS/EAS is supplemented with 50 μM Sulforhodamine B (SRB, Cat#S1402, Sigma-Aldrich), 6 μM monomeric enhanced green fluorescent protein (mEGFP, which was prepared as described in the Supplementary Text S18) or 0.5% w/v 20 nm FluoSpheres Carboxylate-Modified Microspheres (Cat#F8784, Thermo Fisher Scientific). For experiments to investigate the effect of varying temperature, emulsions were prepared at 4 and 20 °C using a 1 mg/mL LiO with an IAS ratio of 2.5%. For the effect of crowding agents, the IAS used for formation was supplemented with 3, 4, and 5% w/v PEG 8000 (Cat#043443.A3, Thermo Fisher Scientific), and sucrose concentrations were lowered to maintain osmolarity. GUVs were made with a 2 mg/mL LiO with an IAS ratio of 2.5%.

### 3.3 Microplate Surface Preparation

96-well microplate wells (Cat#165305, Thermo Fisher Scientific) were passivated to avoid adhesion and bursting of the GUVs. Wells were precoated in 200 μL 5 mg/mL Bovine Serum Albumin (BSA, Cat#A9647, Sigma-Aldrich) in 1× PBS containing 0.1% Biotin labeled bovine albumin (Bio-BSA, Cat#A8549, Sigma-Aldrich) by incubating for 30 min at 4 *°C*. Wells were washed by removing 150 μL and replacing it with 150 μL of 1× PBS. This was repeated a total of 4 times before 150 μL of 10 ng/μL NeutrAvidin (Cat#31055, Thermo Fisher Scientific) was added and incubated for 30 min at 4 °C. 150 μL was removed and washed with 150 μL of EAS for a total of 5 washes. Plates were stored at 4 °C and were used the same day in experiments.

### 3.4 Confocal Microscopy

Produced GUVs were collected by aspiration of the oil layer and resuspension of the GUV pellet in a treated 96-well plate. GUVs were imaged using a BioTek Cytation C10 Confocal Imaging Reader from Agilent Technologies. Images were acquired at 20× and 40× magnification using a 60 μm spinning disk confocal. The GFP, RFP, and Cy5 channels were utilized to detect fluorescent signals from membrane dyes (shown in Supplementary Figure S7) and encapsulated fluorophores. To allow for the direct comparison of fluorescence intensity, all images were acquired using a fixed gain of 3, and pixel intensities were normalized using the integration time for each image to a standard of 25 ms. To correct for nonlinear detectors, a correction factor was applied (shown in Supplementary Figure S1). Flat field corrections were also applied to images to remove unequal illumination (shown in Supplementary Figure S2). Both TIF and PNG images were saved for processing and analysis. For each experimental condition, independent experiments were repeated between 3 and 5 times, and GUVs were imaged and analyzed using QuantGUV.

### 3.5 QuantGUV Image Analysis

Image analysis was performed using a custom graphical user interface (GUI) developed in Python, QuantGUV, available on Zenodo (10.5281/zenodo.17264460), and future updates available on Github (https://github.com/Marshall-Zak/QuantGUV). The program was designed to automate the high-throughput detection and intensity quantification of GUVs from confocal TIF images.

Our analysis pipeline consisted of several steps. First, images were preprocessed. Each image was converted to an 8-bit grayscale format, inverted, and then thresholded using Otsu’s thresholding method (cv2.THRESH_OTSU). Potential GUVs were identified in the resulting binary image using a blob detection algorithm implemented with (cv2.SimpleBlobDetector_create­(params)). The blob detection algorithm isolates groups of bright pixels on a dark background and was used to detect potential GUVs containing fluorescent dyes. Detected blobs were then filtered based on defined parameters for area, circularity, and convexity. Detected objects were filtered to include only those with a size between 5 and 100 μm, a convexity greater than 0.9, and a circularity greater than 0.7. QuantGUV was first validated on user-generated images (shown in Supplementary Figure S3). A circularity greater than 0.7 was applied to exclude objects with irregular or blurred boundaries. For each GUV that fits the parameters specified, a 10 × 10 square pixel region of interest (ROI) was defined at the vesicle’s geometric center. This ensures the mean measurement is representative of the vesicle lumen and minimizes signal from potential membrane interactions. The detected GUVs and the defined ROIs were then quantified from the original unchanged TIF image containing the original observed dynamic range of pixel intensity.

Background correction was applied on an image-by-image basis. A binary mask of all detected GUVs in an image was created, inverted, and assigned as the background area (shown in Supplementary Figure S4). The mean intensity of the lowest valued 10% of pixels in this background region was calculated and subtracted from each GUV’s measured intensity. This method corrects for background noise and out-of-focus fluorescence while avoiding bias from bright aggregates and undetected, out-of-focus vesicles. Following automated analysis, an interactive review step within the GUI allowed for the manual inspection and exclusion of any remaining false positive detections before final data export. The final background-corrected intensity for each GUV was then interpolated on a standard curve to estimate internal concentration.

### 3.6 QuantGUV: Standard Curve Production

Bulk solutions of target fluorescent molecules were prepared in IAS and EAS. For bulk dye, 100 μL of IAS containing a concentration range of the fluorophores was used. Samples were placed into a 96-well microplate and imaged on a Cytation C10 confocal plate reader, with Gain and integration time fixed to 3 and 25 ms. Sample images were collected from multiple points in the well at a *z* height relevant to where the GUVs were observed. For our standard curve, the z value was set to 1900 μm. The mean average of all images in each fluorophore's data set was used as the *I*_Bulk_ value for a given concentration.

For blank GUVs, GUVs were produced as described above in the absence of dye, GUVs were collected as before and resuspended in 100 μL of EAS containing a concentration range of fluorophores. Samples were placed into a 96 well microplate well and imaged on a Cytation C10 confocal plate reader. Gain and integration time were again fixed at 3 and 25 ms, respectively. Images of the blank GUVs were analyzed via QuantGUV using inverted TIFs to detect the blank GUVs as they appear as dark spots on a bright background. The same pipeline was used to detect and quantify intensity within the blank GUVs; however, (cv2.equalizeHist­(image)) and (cv2.GaussianBlur­(equalized_image, (11, 11), 0)) were used to enhance the contrast of images further to enable detection at low fluorophore concentrations. The internal signal of the blank GUVs was assigned to the *I*_Blank_ value and the background signal was assigned to the *I*_Bulk_ for the purpose of determining *I*_Inside_ for each GUV.

The calculated *I*_Inside_ values were plotted with the relevant concentration of fluorescent molecule to create standard curves (shown in Supporting Information Figure S5). for each molecule, blank IAS and EAS were used to sample a zero fluorescence condition to which the intercept of each standard curve was set.

To quantify the fluorescence enhancement of mEGFP caused by molecular crowding, the bulk fluorescence of 6 μM mEGFP was measured in the presence of 0, 3, 4, and 5% (w/v) PEG 8000. A correction ratio was calculated Ratio = Intensity_PEG_/Intensity_Control_ and applied to the mEGFP standard curve to generate condition-specific calibration slopes.

To ensure longitudinal consistency and account for potential batch-to-batch variability in fluorophore properties, it is recommended that a new standard curve be generated for each new lot of target molecules. This step ensures that the QuantGUV pipeline remains calibrated to the specific optical signature of the current reagent batch.

### 3.7 Statistical Analysis

Statistical analysis was conducted using R Studio Build 513. To identify significant differences in GUV encapsulated concentrations at different LiO Concentrations (Figure 3B), a one-way ANOVA was carried out in R Studio, followed by a Tukey’s posthoc test (*p* < 0.05) to identify groups of significant difference. For identifying significant differences in GUV encapsulated concentrations at different LiO Concentrations as IAS ratios (Figure 4), a two-way ANOVA was carried out in R Studio, followed by a Tukey’s posthoc test (*p* < 0.05) to identify groups of significant difference. Significance was represented in Figures 3B and 4 with the convention of letters (a–f) where columns that share one letter the same letter are not significantly different from each other, whereas if no common letters are shared, a significant difference was observed.

For identifying significance in the effect of PEG concentration on encapsulation efficiency (Figure 5A), a one-way ANOVA was carried out in R Studio, followed by a Tukey’s posthoc test (*p* < 0.05) to identify groups of significant difference. Significance was represented in Figure 5A with the convention of letters (a, b, and c) where columns that share one letter the same letter are not significantly different from each other, whereas if no common letters are shared, a significant difference was observed.

For identifying significance in the effect of temperature on encapsulation efficiency, a two-tailed independent *t*-test (Welch’s *t*- test) was conducted, with a threshold alpha of 0.05. Significance was shown with significance brackets with *p* values being represented in Figure 5B using the convention of * indicates *p* < 0.05, ** indicates *p* < 0.01, *** indicates *p* < 0.001, and **** indicates *p* < 0.0001.

## 4 Discussion

In this work, we developed QuantGUV, a standardized, accessible software pipeline to quantify concentrations of fluorescent molecules encapsulated in GUVs at the single GUV level. By applying this high-throughput method, we systematically investigated how various factors, including physical properties of molecules, lipid concentration, IAS ratio, temperature, and addition of crowding agents, influence the encapsulation efficiency during the GUV formation. Our findings reveal that encapsulation of molecules inside GUVs is not governed by a single parameter but is a complex interplay of factors, with molecular size being a particularly critical determining factor.

### 4.1 Molecular Size and Lipid Concentration Dictate Encapsulation Success

The size of the molecules has the biggest impact on the encapsulation efficiency, with a higher yield for bigger molecules. This is expected as small defects in the lipid membrane that could form during the transfer or centrifugation process would not be sufficiently large to allow bigger molecules to leak as readily. There is also indirect evidence that bigger molecules have a higher encapsulation efficiency. As with the PURE expression system, only with the addition of small molecules in the external environment outside of the GUVs were there sufficient building blocks internally to achieve high yields for biochemical reactions.^39^

In addition, 20 nm particles diffuse significantly more slowly than molecules of 1 nm in size. We attribute the negative correlation between LiO concentration and encapsulation efficiency for SRB and FluoSpheres to their apparent membrane affinity. Unlike mEGFP, both SRB and FluoSpheres accumulate at the membrane, creating a distinct “halo” effect (see Supplementary Figure S8). This membrane affinity is consistent with a concentration depletion within the aqueous core that appears to scale with lipid availability. Crucially, because QuantGUV is explicitly programmed to extract intensity solely from a 10 × 10 pixel ROI at the geometric center of the vesicle, it precisely reports this exacerbated internal depletion. When the LiO concentration is increased, the total capacity of this lipid “sink” is magnified, causing a larger fraction of encapsulated SRB or FS to partition out of the central aqueous lumen and into the membrane boundary. In contrast, mEGFP is highly hydrophilic and does not exhibit this membrane sequestration behavior, allowing it to maintain higher internal concentrations as lipid density increases.

### 4.2 The Influence of IAS Ratio on Encapsulation Efficiency

For SRB, as the IAS increases within a low-lipid-concentration sample, the efficiency increases. We believe this is due to a lower ratio of lipid to aqueous solution, which means that for each droplet, the apparent lipid availability per droplet decreases and lowers the probability that SRB will partition into the membrane. This, in turn, means more SRB in the lumen and a higher encapsulation efficiency observed, whereas in a high lipid concentration sample, as the IAS ratio increases, the same lipid-to-aqueous solution ratio does not increase as drastically, and therefore, the sink effect is more constant. For example, 0.5 mg/mL LiO efficiency increases from 74 to 126% as IAS increases, whereas at 5 mg/mL LiO, efficiency increases less drastically from 36 to 46% as IAS increases. This represents a 70% relative change compared with a 27% change for higher lipid concentrations. However, mixed results were observed for other molecules, and therefore, we cannot attribute this behavior to the effect of the size of molecules.

### 4.3 Effect of Varied Temperature and Crowding Agents on Encapsulation

The significant increase in mEGFP encapsulation efficiency, rising from 58.73% under PEG-free conditions to 107.27% at 5% w/v PEG 8000, highlights the importance of the internal physical environment during vesicle formation. We attribute this enhancement to the synergistic interplay of an increased internal viscosity and the excluded volume effect. In the water-in-oil emulsion transfer method, GUVs are formed as aqueous droplets in an oil phase that cross the interface between the immiscible water and the oil phases. This phase transfer is a mechanically stressful event, where transient pores can spontaneously occur in the developing lipid bilayer. By incorporation of PEG-8000, the elevated viscosity of the IAS could slow the translational diffusion of the fluorescent molecules. This may act as a kinetic trap, potentially reducing the rate of leakage through transient membrane defects before the bilayer is fully sealed. In addition, the excluded volume effect exerted by the PEG polymers provides an entropic drive that reduces the available solvent space for mEGFP. This effect likely crowds the protein away from the membrane interface, further minimizing the probability of escape through transient pores.

In contrast, our results show that temperature appears to exert an opposing influence. Reducing the formation temperature from 20 to 4 °C resulted in a statistically significant decrease in mean encapsulation efficiency from 52.6 to 40.8%. This reduction in yield stems from both lipid thermodynamics and the temperature-dependent viscosity of the oil phase. At lower temperatures, increased ordering of the lipid acyl chains reduces membrane fluidity, slowing the repair of transient defects. Furthermore, the higher viscosity of the oil phase increases drag during centrifugation, prolonging the time droplets spend in a fragile state at the oil–water interface and thereby increasing leakage. Despite this reduction in encapsulation efficiency, maintaining a low temperature of 4 °C is a necessary strategic trade-off. The preparation and mixing process typically requires approximately 30 min. Conducting this step at 4 °C is essential to stall biological reactions and prevent the premature initiation of encapsulated biochemical systems. Therefore, while higher temperatures favor encapsulation yield, lower temperatures are preferred to preserve the functional integrity of the synthetic cell systems.

### 4.4 Interpretation of Supernominal Encapsulation Efficiencies

Efficiencies exceeding 100% were observed under some conditions for all three molecules. One possible explanation is the formation of small, transient defects that permit water efflux while retaining the encapsulated molecules, although we cannot directly validate this mechanism here. The concentrating effect was more pronounced for larger molecules at high concentrations, similar to previous studies on macromolecular encapsulation. Notably, Baldauf et al. reported ‘superconcentration’ of actin encapsulated within GUVs reaching up to 1.7-fold of the nominal value.^19^

Furthermore, we evaluated the diameters of GUVs detected against their encapsulation efficiency to determine if there is a correlation between size and encapsulation of GUVs. While there was a weak positive correlation for certain conditions between encapsulation and size, it is not possible to generalize or predict trends from this data set. (shown in Supporting Information Figure S17)

### 4.5 Methodological Robustness and Limitations

QuantGUV addresses several shortcomings of established luminal quantification techniques, such as fluorescence correlation spectroscopy (FCS), which is typically constrained by low concentration ranges and low throughput. To ensure the robustness of our data, we incorporated several safeguards against optical and physical artifacts.

To account for light scattering by lipid membranes, the pipeline utilizes blank GUV populations prepared via the same formation method and exhibiting similar size distributions as experimental samples. Additionally, GUVs of different sizes sequestered at the surface of the microplate may have differing *z* locations for their equatorial planes. To ensure measurements are taken from the equatorial plane, QuantGUV applies a circularity filter:

$$\mathrm{Circularity}=\frac{4\pi \times \mathrm{Area}}{{\mathrm{Perimeter}}^{2}}$$

By mathematically excluding objects with a circularity score below 0.7, the software rejects vesicles imaged at nonequatorial planes where the membrane appears blurred or thickened. Residual geometric errors are further minimized by measuring intensity only from a defined 10 × 10 ROI at the geometric center of a detected circumference, which prevents interference from the membrane boundary and ensures the quantification is mathematically immune to size-based intensity bias.

A fundamental constraint of intensity-based measurement is the potential for quenching and photobleaching. We mitigated these by conducting all quantifications within the empirically established linear dynamic range for each molecule, where self-quenching effects were shown to be negligible. All quantification data were acquired under consistent, standardized laser and gain settings to protect fluorophore integrity. While GUVs are intrinsically subject to stochastic leakage and volume fluctuations, QuantGUV provides a “snapshot” measurement of the final steady-state internal concentration. This approach ensures that reported efficiencies reflect the actual concentration available for biochemical reactions at the time of imaging, inherently accounting for preceding physical content loss. Crucially, this explains why some populations (e.g., FluoSpheres) exhibit apparent encapsulation efficiencies exceeding 100%; these values reflect physical phenomena like preferential oil evaporation rather than algorithmic errors. Finally, because our standard curve calibration accounts for out-of-focus light, this methodology could theoretically be adapted for standard wide-field microscopy. Importantly, QuantGUV is optimized for relative and comparative encapsulation analysis across conditions; absolute encapsulation efficiencies may still depend on preparation-specific variables inherent to emulsion-transfer GUV systems, such as oil composition, residual solvent, and operator handling.

### 4.6 Future Outlook and Broader Impact

As QuantGUV relies on user-defined standard curves, it is independent of specific lipid compositions and can be applied to a wide variety of synthetic-cell systems. By offering a low barrier to entry, this tool enables the shift from trial-and-error assembly toward systematically engineered synthetic cells with predictable internal environments. Such precision is critical for the accurate mathematical modeling of complex cellular networks.

Future work should explore a broader range of membrane compositions, including the incorporation of cholesterol or charged lipids, and the coencapsulation of multiple functional components. While our current validation relies on internal bulk standards, future studies could provide independent verification of luminal concentrations by coupling QuantGUV with orthogonal methods, such as microfluidic-based assays or flow cytometry.

## 5 Conclusions

In this study, we introduced QuantGUV, an accessible, high-throughput software pipeline for quantifying the encapsulation efficiency of fluorescent molecules in GUVs at single-vesicle resolution. By applying this tool, our findings demonstrate that encapsulation is not governed by a single parameter but by a complex interplay of physical and chemical factors, with molecular size and lipid concentration playing particularly critical roles. Furthermore, we established that environmental conditions during formation, such as temperature and the presence of macromolecular crowding agents, significantly influence the final luminal concentrations. By addressing the low-throughput and resolution limitations of existing quantification techniques, QuantGUV provides a standardized, low-barrier-to-entry tool that is independent of specific lipid composition. This capability enables researchers to move beyond trial-and-error assembly toward the systematic engineering of synthetic cells with accurately predicted internal environments. Future research will focus on applying QuantGUV to a broader range of membrane compositions, including charged lipids and cholesterol, and exploring the coencapsulation of multiple functional components. Additionally, future studies can strengthen this validation by coupling QuantGUV analysis with orthogonal quantification methods, such as microfluidic-based assays or flow cytometry.

## Supplementary Material



## Notes

QuantGUV calibration and test data, limits of quantification, merged channel images, halo effect analysis, confocal images for all tested experimental conditions, data distributions for encapsulation efficiency and GUV diameters, expanded analyses of PEG-8000 and temperature effects, statistical analysis data summaries, linear regression analysis of size versus encapsulation efficiency and GFP production protocol (PDF)

## References

[1] Das S., Patki G. M., Sridhar V., Mulewar S., Roy R., Bandyopadhyay U., Kulshreshtha N., Rajamani S. (2024). The european physical
journal special topics compartmentalization as a ubiquitous feature
of life: from origins of life to biomimetics. Eur. Phys. J. Spec. Top.

[2] Giessen T. W., Silver P. A. (2016). Encapsulation as a strategy for the design of biological
compartmentalization. J. Mol. Biol..

[3] Tivony R., Fletcher M., Al Nahas K., Keyser U. F. (2021). A microfluidic platform
for sequential assembly and separation of synthetic cell models. ACS Synth. Biol..

[4] Ip T., Li Q., Brooks N., Elani Y. (2021). Manufacture of multilayered
artificial
cell membranes through sequential bilayer deposition on emulsion templates. ChemBioChem.

[5] Wagner A. M., Quandt J., Söder D., Garay-Sarmiento M., Joseph A., Petrovskii V. S., Witzdam L., Hammoor T., Steitz P., Haraszti T., Potemkin I. I., Kostina N. Y., Herrmann A., Rodriguez-Emmenegger C. (2022). Ionic combisomes:
A
new class of biomimetic vesicles to fuse with life. Adv. Sci..

[6] Shin J., Cole B. D., Shan T., Jang Y. (2022). Heterogeneous synthetic
vesicles toward artificial cells: Engineering structure and composition
of membranes for multimodal functionalities. BioMacromolecules.

[7] Moga A., Yandrapalli N., Dimova R., Robinson T. (2019). Optimization of the
inverted emulsion method for high-yield production of biomimetic giant
unilamellar vesicles. ChemBioChem.

[8] Pautot S., Frisken B. J., Weitz D. A. (2003). Production
of unilamellar vesicles
using an inverted emulsion. Langmuir.

[9] Van
De Cauter L., Fanalista F., Van Buren L., De Franceschi N., Godino E., Bouw S., Danelon C., Dekker C., Koenderink G. H., Ganzinger K. A. (2021). Optimized
cdice for efficient reconstitution of biological systems in giant
unilamellar vesicles. ACS Synth. Biol..

[10] Van
de Cauter L., van Buren L., Koenderink G. H., Ganzinger K. A. (2023). Exploring giant unilamellar vesicle production for
artificial cells—current challenges and future directions. Small Methods.

[11] Salehi-Reyhani A., Ces O., Elani Y. (2017). Minireview artificial cell mimics as simplified models
for the study of cell biology impact statement. Exp. Biol. Med..

[12] Walde P., Cosentino K., Engel H., Stano P. (2010). Giant vesicles: Preparations
and applications. ChemBioChem.

[13] Uzun H. D., Tiris Z., Czarnetzki M., López-Marqués R. L., Pomorski T. G. (2024). Electroformation
of giant unilamellar vesicles from
large liposomes. Eur. Phys. J. Spec. Top..

[14] Shimane Y., Kuruma Y. (2022). Rapid and facile preparation
of giant vesicles by the
droplet transfer method for artificial cell construction. Front. Bioeng. Biotechnol..

[15] Matsushita-Ishiodori Y., Hanczyc M. M., Wang A., Szostak J. W., Yomo T. (2019). Using imaging
flow cytometry to quantify and optimize giant vesicle production by
water-in-oil emulsion transfer methods. Langmuir.

[16] Stachowiak J. C., Richmond D. L., Li T. H., Liu A. P., Parekh S. H., Fletcher D. A. (2008). Unilamellar vesicle formation and
encapsulation by
microfluidic jetting. Proc. Natl. Acad. Sci.
U.S.A..

[17] Tsugane M., Suzuki H. (2020). Elucidating the membrane dynamics and encapsulation
mechanism of large dna molecules under molecular crowding conditions
using giant unilamellar vesicles. ACS Synth.
Biol..

[18] Nair K. S., Raj N. B., Madhavan
Nampoothiri K., Mohanan G., Acosta-Gutiérrez S., Bajaj H. (2022). Curved membrane structures
induced by native lipids in giant vesicles. J. Colloid Interface Sci..

[19] Baldauf, L. ; Frey, F. ; Perez, M. A. ; Mladenov, M. ; Way, M. ; Idema, T. ; Koenderink, G. H. Biomimetic actin cortices shape cell-sized lipid vesicles. bioRxiv 2023. 10.1101/2023.01.15.524117.

[20] Kanwa, N. ; Reverte-López, M. ; Schwille, P. In vitro reconstitution of cytoskeletal networks inside phase separated giant unilamellar vesicles (guvs). J. Vis. Exp. 2025, e68530 10.3791/68530. 40622871

[21] Monck C., Elani Y., Ceroni F. (2024). Genetically programmed synthetic
cells for thermo-responsive protein synthesis and cargo release. Nat. Chem. Biol..

[22] Jahnke K., Huth V., Mersdorf U., Liu N., Göpfrich K. (2022). Bottom-up
assembly of synthetic cells with a dna cytoskeleton. ACS Nano.

[23] Fletcher M., Zhu J., Rubio-Sánchez R., Sandler S. E., Al Nahas K., Di Michele L., Keyser U. F., Tivony R. (2022). Dna-based optical quantification
of ion transport across giant vesicles. ACS
Nano.

[24] Liang W., Levchenko T. S., Torchilin V. P. (2004). Encapsulation of atp into liposomes
by different methods: optimization of the procedure. J. Microencapsulation.

[25] Miwa A., Kamiya K. (2022). Control of enzyme reaction
initiation inside giant
unilamellar vesicles by the cell-penetrating peptide-mediated translocation
of cargo proteins. ACS Synth. Biol..

[26] Nishimura K., Matsuura T., Nishimura K., Sunami T., Suzuki H., Yomo T. (2012). Cell-free protein synthesis
inside giant unilamellar vesicles analyzed
by flow cytometry. Langmuir.

[27] Soga H., Fujii S., Yomo T., Kato Y., Watanabe H., Matsuura T. (2014). In vitro membrane protein synthesis inside cell-sized
vesicles reveals the dependence of membrane protein integration on
vesicle volume. ACS Synth. Biol..

[28] Smith J. M., Hartmann D., Booth M. J. (2023). Engineering
cellular communication
between light-activated synthetic cells and bacteria. Nat. Chem. Biol..

[29] Dreher Y., Jahnke K., Schröter M., Göpfrich K. (2021). Light-triggered
cargo loading and division of dna-containing giant unilamellar lipid
vesicles. Nano Lett..

[30] Ernits M., Reinsalu O., Yandrapalli N., Kopanchuk S., Moradpur-Tari E., Sanka I., Scheler O., Rinken A., Kurg R., Kyritsakis A., Linko V., Zadin V. (2024). Microfluidic
production, stability and loading of synthetic giant unilamellar vesicles. Sci. Rep..

[31] Lombardo D., Kiselev M. A. (2022). Methods of liposomes
preparation: Formation and control
factors of versatile nanocarriers for biomedical and nanomedicine
application. Pharmaceutics.

[32] Elani Y. (2016). Construction
of membrane-bound artificial cells using microfluidics: a new frontier
in bottom-up synthetic biology. Biochem. Soc.
Trans..

[33] Dolder N., Mü P., Von Ballmoos C. (2022). Experimental platform for the functional
investigation of membrane proteins in giant unilamellar vesicles. Soft Matter.

[34] Dominak L. M., Keating C. D. (2007). Polymer encapsulation
within giant lipid vesicles. Langmuir.

[35] Dominak L. M., Omiatek D. M., Gundermann E. L., Heien M. L., Keating C. D. (2010). Polymeric
crowding agents improve passive biomacromolecule encapsulation in
lipid vesicles. Langmuir.

[36] Schwille P., Spatz J., Landfester K., Bodenschatz E., Herminghaus S., Sourjik V., Erb T. J., Bastiaens P., Lipowsky R., Hyman A., Dabrock P., Baret J. C., Vidakovic-Koch T., Bieling P., Dimova R., Mutschler H., Tom Robinson T. Y., Tang D., Wegner S., Sundmacher K. (2018). Maxsynbio:
Avenues towards creating cells from the bottom up. Angew. Chem., Int. Ed..

[37] Runas K. A., Malmstadt N. (2014). Low levels of lipid oxidation radically increase the
passive permeability of lipid bilayers. Soft
Matter.

[38] Hindley J. W., Elani Y., Mcgilvery C. M., Ali S., Bevan C. L., Law R. V., Ces O. (2018). Light-triggered enzymatic
reactions
in nested vesicle reactors. Nat. Commun..

[39] Garamella J., Marshall R., Rustad M., Noireaux V. (2016). 344–355
downloaded
via univ of surrey on. ACS Synth. Biol..

[40] Gonzales D. T., Yandrapalli N., Robinson T., Zechner C., Tang T.-Y. D. (2022). Cell-free
gene expression dynamics in synthetic cell populations. ACS Synth. Biol..

[41] Sun B., Chiu D. T. (2005). Determination of
the encapsulation efficiency of individual
vesicles using single-vesicle photolysis and confocal single-molecule
detection. Anal. Chem..

[42] Matosevic S., Paegel B. M. (2011). Stepwise synthesis of giant unilamellar vesicles on
a microfluidic assembly line. J. Am. Chem. Soc..

[43] Göpfrich K., Haller B., Staufer O., Dreher Y., Mersdorf U., Platzman I., Spatz J. P. (2019). One-pot
assembly of complex giant
unilamellar vesicle-based synthetic cells. ACS
Synth. Biol..

[44] Liu Y., Zhang M., Zhao J., Ren Y., Li S., Wang W., Wei M., Han X. (2025). Construction
of a de
novo nucleotide biosynthesis pathway in artificial cells for rna transcription. J. Am. Chem. Soc..

[45] Supramaniam P., Wang Z., Chatzimichail S., Parperis C., Kumar A., Ho V., Ces O., Salehi-Reyhani A. (2023). Measuring encapsulation efficiency
in cell-mimicking giant unilamellar vesicles. ACS Synth. Biol..

[46] Noireaux V., Libchaber A. (2004). A vesicle bioreactor as a step toward
an artificial
cell assembly. Proc. Natl. Acad. Sci. U. S.
A..

[47] Bae W., Yoon T. Y., Jeong C. (2021). Direct evaluation
of self-quenching
behavior of fluorophores at high concentrations using an evanescent
field. PLoS One.

[48] Muhammad N., Kryuchkova N., Dworeck T., Rodríguez-Ropero F., Fioroni M. (2013). Enhanced egfp fluorescence emission in presence of
peg aqueous solutions and pib1000-peg6000-pib1000 copolymer vesicles. BioMed. Res. Int..

