# Supplementary material for: QuantGUV: Quantifying Encapsulation Efficiency of Small Molecules in Giant Unilamellar Vesicles
Source: ACS Appl Mater Interfaces. 2026 May 28;18(22):30863–73. doi: 10.1021/acsami.6c03651 (PMC13266705; doi:10.1021/acsami.6c03651)
Supplement: Supplementary file 1 [file am6c03651_si_001.pdf]

Supporting Information

QuantGUV: Quantifying Encapsulation Efficiency of Small Molecules in Giant Unilamellar Vesicles

Zak Marshall[1], Reshma Bano[1], Pasha Dylan[1], Luisa Trifan[1], Callum Mckeaveney[2], André P. Gerber[2] and Wooli Bae[1,*]

[1] School of Mathematics & Physics, Faculty of Engineering and Physical Sciences, University of Surrey, Surrey GU2 7XH, United Kingdom
[2]School of Biosciences, FHMS, University of Surrey, Surrey GU2 7XH, United Kingdom
Corresponding Author: w.bae@surrey.ac.uk

Contents:

QuantGUV Calibration and Tests	Figures S1-S5 and Table S6
Merged Channels, DiD and each fluorophore	Figure S7
Halo effect at GUV membrane	Figure S8
GUV images at all conditions in figure 4	Figure S9
Heterogeneity of encapsulation efficiency in figure 4	Figure S10
GUV diameter distribution in figure 4	Figure S11
Effect on Encapsulation expanded: PEG-8000 and Temperature	Figure S12
Statistical Analysis Data	Tables S13-S16
Linear regression of Size vs Encapsulation efficiency	Figure S17
GFP Production Protocol	Text S18

Figure S1: integration time is an intensity per pixel per second factor, allowing for the normalization by the division of the integration time used and multiplied by 25 to achieve consistency with the standard curve. However detectors are non linear so a minor adjustment must be made to account for this effect

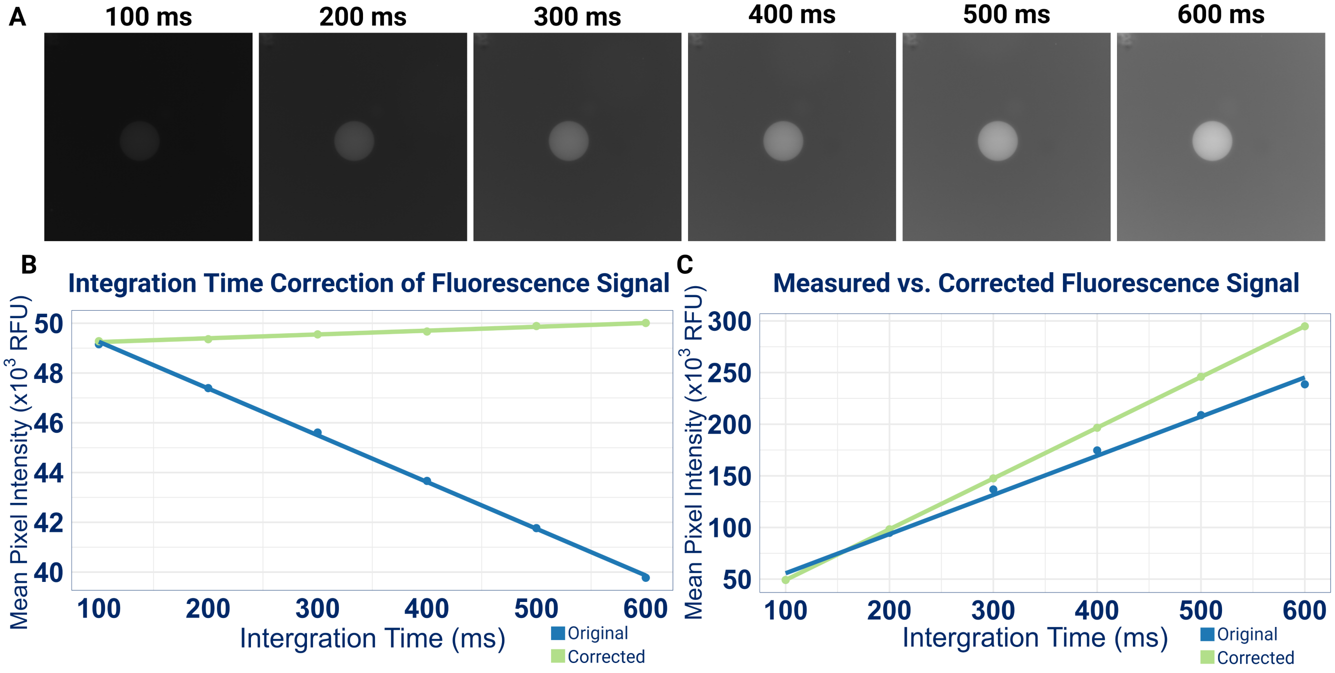

Figure S1: Integration time correction of fluorescence intensity A a time series of a GUV images as integration time increases B Pixel intensity was linearised through an empirical correction factor generated using integration times in a Polynomial Regression C Empirical correction at non normalised intensities showing linearisation of detected signal

Figure S2: Detected images were flattened using a flat fielding correction factor applied to all images before quantification.

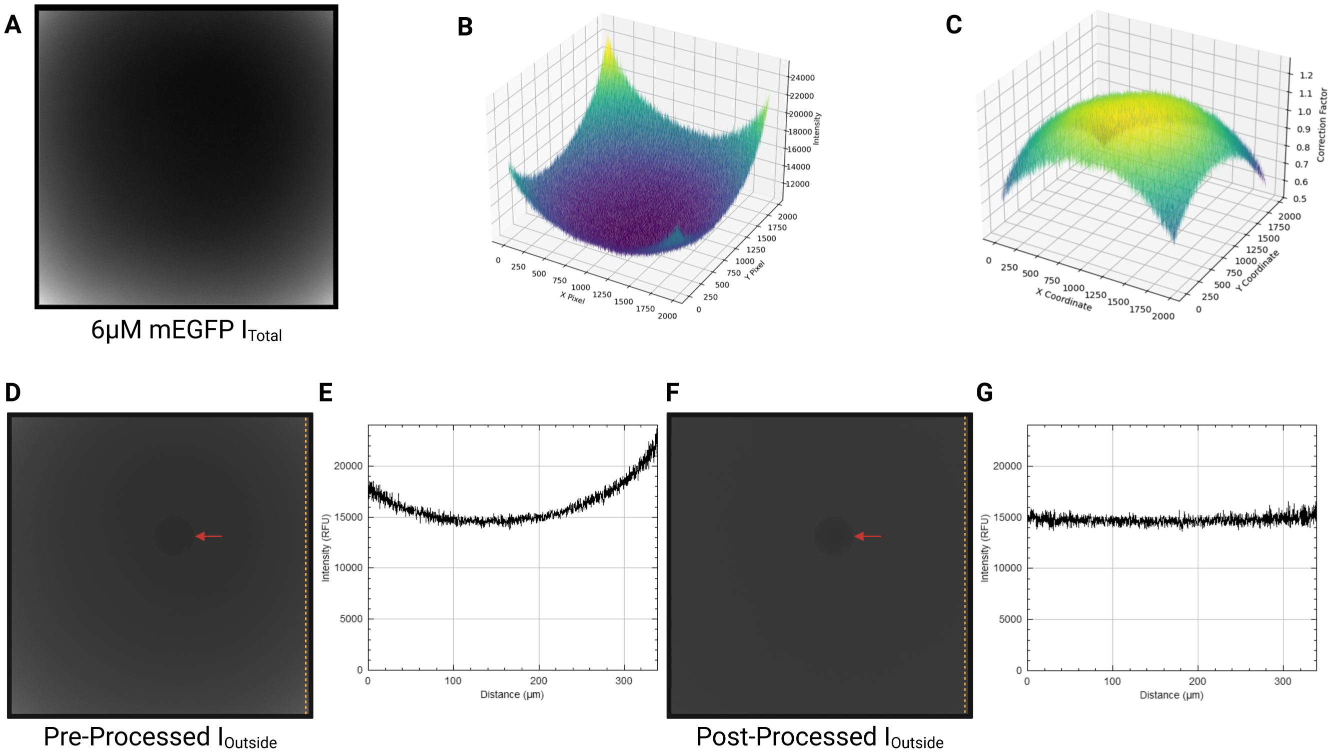

Figure S2: Detector normalisation through flat field processingA Bulk images of each fluorescent dye at max concentration were obtained, Tif placed on Black square to highlight vignette from detector B 3D pixel mapping to inspect vignette observed C 3D coordinate map of correction factors, produced from taking a mean pixel intensity from B and applying a factor to flatten the image D Pre-Processed $I_{Outside}$ ***image, Red arrow indicated a blank GUV, yellow dashed line indicates location of intensity profile plot E Intensity profile plot for D F Post-Processed*** $I_{Outside}$ ***image, Red arrow indicated a blank GUV, yellow dashed line indicates location of Intensity profile plot G Intensity profile plot for F***

Figure S3: Test images were generated using python code to produce circles of varying size, circularity and intensity on a background intensity of users choice to test the calculations of QuantGUV on known parameters

A

```
# --- Image Settings ---
IMAGE_WIDTH = 1960
IMAGE_HEIGHT = 1960
BACKGROUND_INTENSITY = 5000
circles_properties = [
    # GUV1:
    {'radius_y': 28.9,  'radius_x': 28.9,  'intensity': 25000},
    # GUV2:
    {'radius_y': 57.8,  'radius_x': 57.8,  'intensity': 30000},
    # GUV3:
    {'radius_y': 86.7,  'radius_x': 86.7,  'intensity': 35000},
    # GUV4:
    {'radius_y': 115.6, 'radius_x': 115.6, 'intensity': 40000},
    # GUV5:
    {'radius_y': 144.5,  'radius_x': 144.5,  'intensity': 45000},
```

B

GUV ID	Diameter (μm)	Intensity	Background Normalised
GUV 1	10	25000	20000
GUV 2	20	30000	25000
GUV 3	30	35000	30000
GUV 4	40	40000	35000
GUV 5	50	45000	40000

C **D**

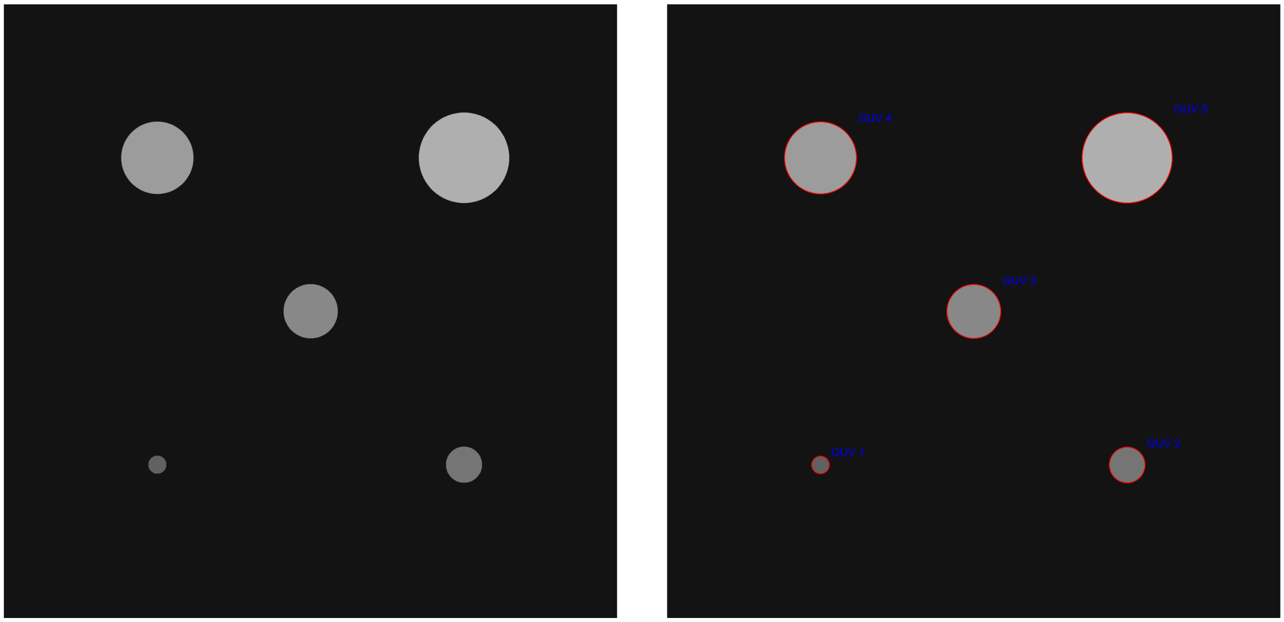

Figure S3: QuantGUV test image calibration A Custom code determining parameters of test images B Output from QuantGUV indicating correct calculations and subtractions were performed C Test TIF created an provided to QuantGUV D Output PNG from QuantGUv showing "GUV" detection

Figure S4: Image per Image background correction is built into QuantGUV, a mask of the detected vesicles that fit a parameter is produced and inverted to create a background mask, isolating all pixels associated with the background.

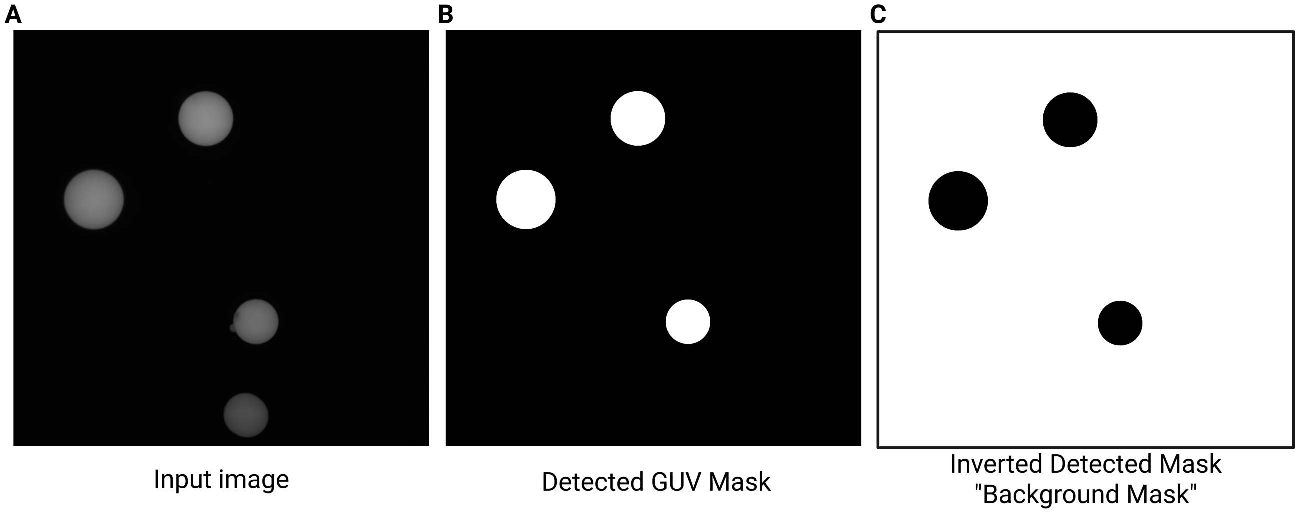

Figure S4: Background mask generationA Input TIF image provided to QuantGUV B Output PNG mask of detected GUVs C Output PNG of background mask, inverted from B

Figure S5: Standard curves were produced as described in main text, a standard curve was produced for each magnification to eliminate variation in intensity caused by magnification differences in data sets allowing for more insights at the individual level and population level for all conditions

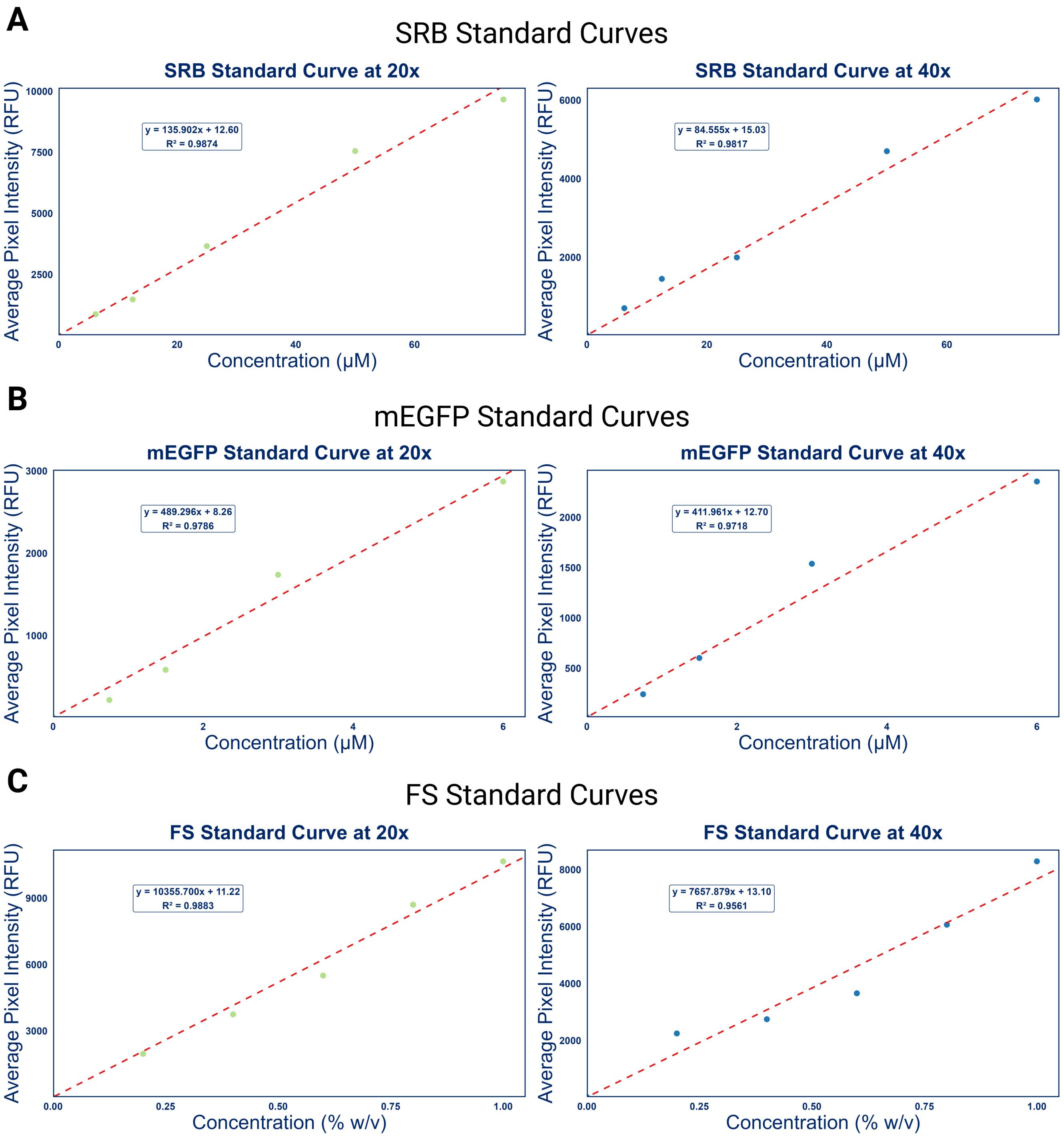

Figure S5: Standard Curves for fluorophores at each magnificationA SRB 20x Standard curve B SRB 40x Standard curve C mEGFP 20x Standard curve D mEGFP 40x Standard curve E FS 20x Standard curve F FS 40x Standard curve

Table S6: Limits of quantification for the standard curve were analysed using line data and LOQ determined for each condition.

Limits of Quantitation (LOQ) for Selected Fluorophores

Fluorophore	Mag.	Slope (RFU/unit)	Intercept (RFU)	R2	Linear Range	Min. Quantifiable Conc. (LOQ)
SRB	20x	135.9	12.6	0.987	5 – 75 µM	67.6 nM
mEGFP	20x	489.3	8.3	0.979	0.5 – 6 µM	19.2 nM
FluoSpheres	20x	10355.7	11.2	0.988	0.2 – 1.0 %	8.87×10^{-4} %
SRB	40x	84.5	15	0.981	5 – 75 µM	36.2 nM
mEGFP	40x	411.9	12.7	0.971	0.5 – 6 µM	29.5 nM
FluoSpheres	40x	7657.8	13.1	0.956	0.2 – 1.0 %	4.00×10^{-4} %

Table S6: Limit of Quantification analysis Concentrations for SRB and mEGFP are reported in nanomolar (nM), while FluoSpheres are reported as a volume percentage (%). R^2 values represent the coefficient of determination for the linear regression. LOQ was calculated using the formula $LOQ = \frac{10\times\sigma}{S}$, where σ is the standard deviation of the y-intercept and S is the slope.

Figure S7: DiD was added to all LiO preparations to allow for screening of GUVs, Merged Images were collected to show internal volume signal as well as membrane bound signal.

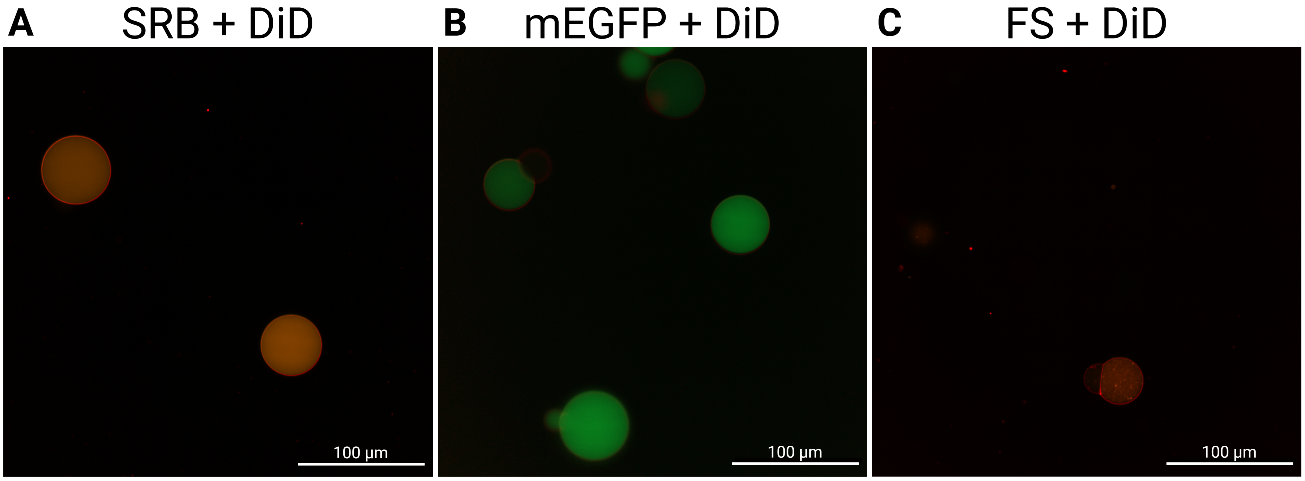

S7: Merged GUV ImagesA Merged GUV image of DiD and SRB B Merged GUV image of DiD and mEGFP C Merged GUV image of DiD and FS. Scale bars = 100 µm

Figure S8: A Halo Effect was observed in some conditions of SRB and FS, Intensity profile plots were used to inspect and visualise this effect at the membrane of GUVs and its absence in mEGFP

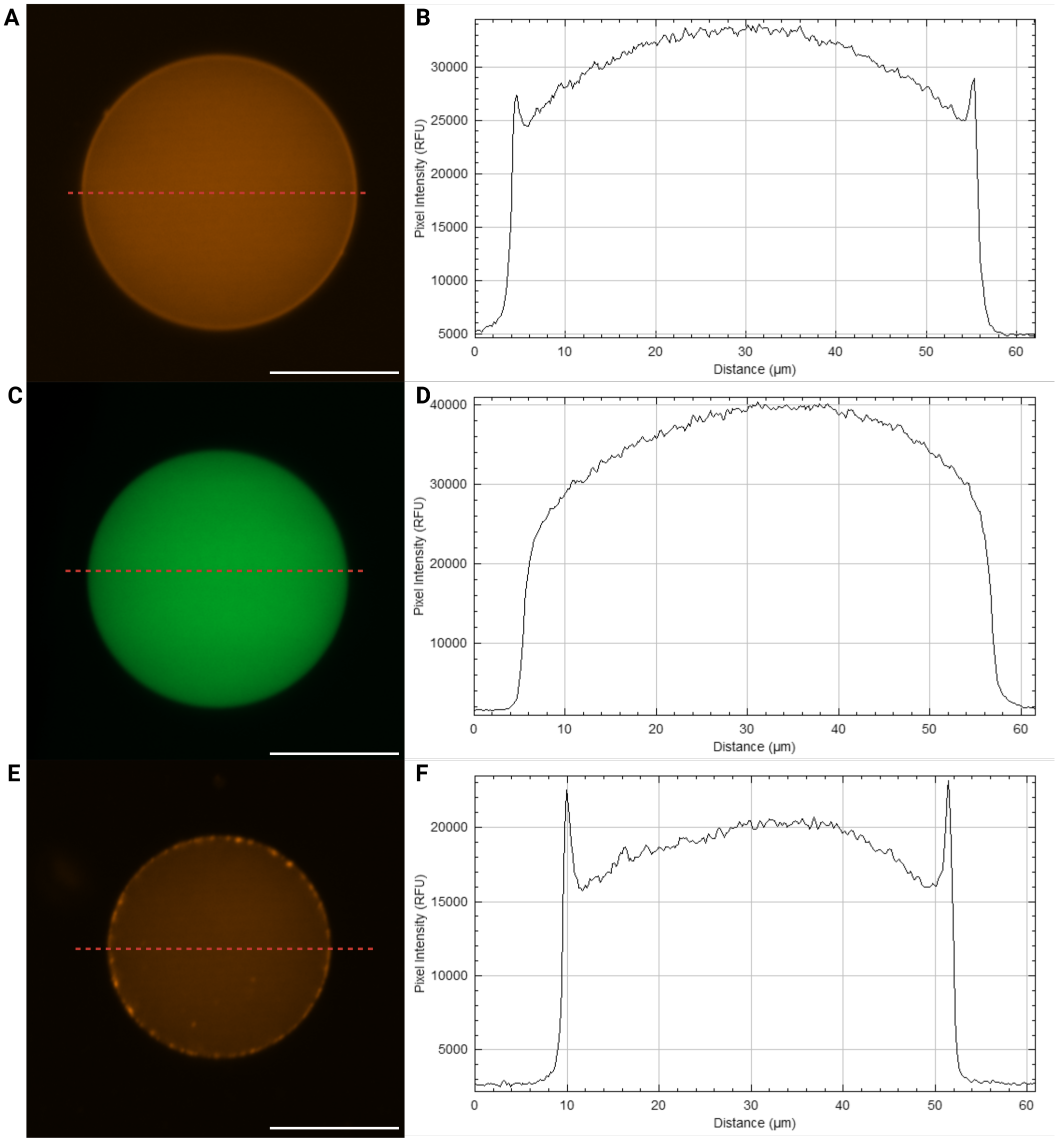

Figure S8: Halo Effect in GUVs containing SRB and FSA Confocal image of GUV containing SRB with a halo effect at the membrane, Red line represents the location of Intensity profile plot scale bar* = 25 µM *B Intensity profile plot of A, with boundary fluorescence indicating the Halo effect in the cross section of GUV C Confocal image of GUV containing mEGFP without a halo effect at the membrane, Red line represents the location of Intensity profile plot scale bar* = 25 µM *D Intensity profile plot of C, without boundary fluorescence indicating supporting no Halo effect in the cross section of GUV E Confocal image of GUV containing FS with a halo effect at the membrane, Red line represents the location of Intensity profile plot scale bar* = 25 µM *F Intensity profile plot of E, with boundary fluorescence indicating Halo effect in the cross section of GUV

Figure S9: Representative confocal images of each condition tested in figure 4, containing a matrix of LiO concentrations and IAS ratio per fluorescent molecules

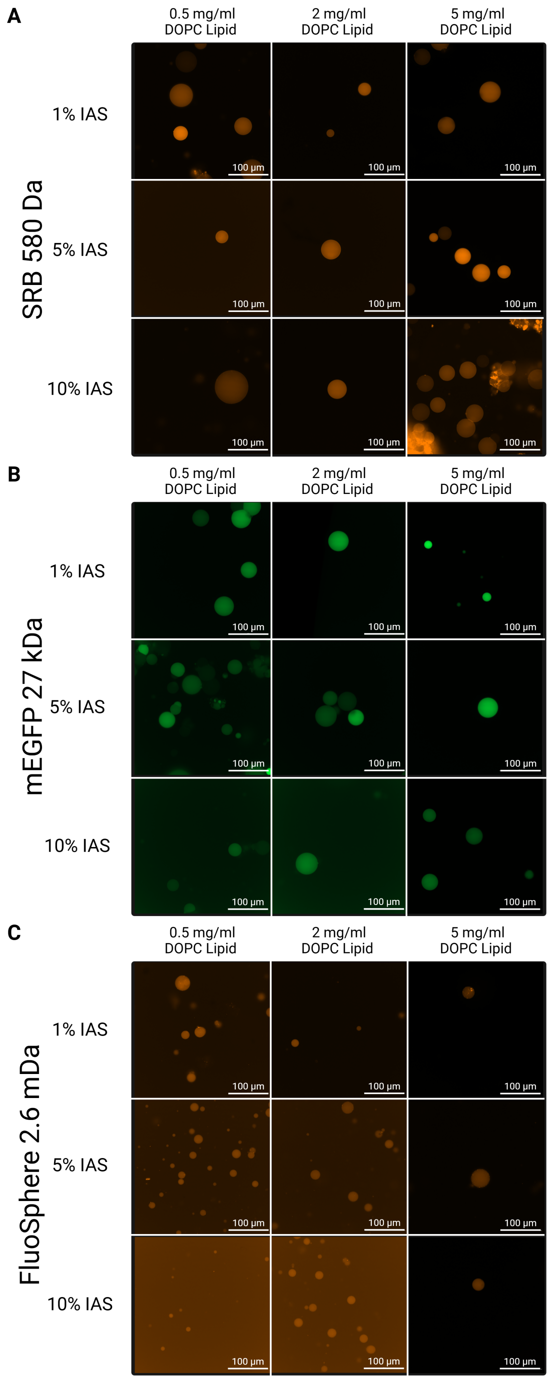

Figure S9: Confocal images of GUVs in conditions in figure 4A SRB confocal image matrix of GUVs containing SRB 580 Da at LiO concentrations of 0.5, 2 *and* 5 *mg/ml* ***in combination with IAS ratios of*** 1, 5 *and* 10% ***B mEGFP confocal image matrix of GUVs containing mEGFP 27 kDa at LiO concentrations of*** 0.5, 2 *and* 5 *mg/ml* ***in combination with IAS ratios of*** 1, 5 *and* 10% ***C FS confocal image matrix of GUVs containing FS 2.6 MDa at LiO concentrations of*** 0.5, 2 *and* 5 *mg/ml* ***in combination with IAS ratios of*** 1, 5 *and* 10%

Figure S10: Raincloud plots displaying the distribution of Encapsulation efficiency of each condition tested in figure 4, containing a matrix of LiO concentrations and IAS ratio per fluorescent molecules

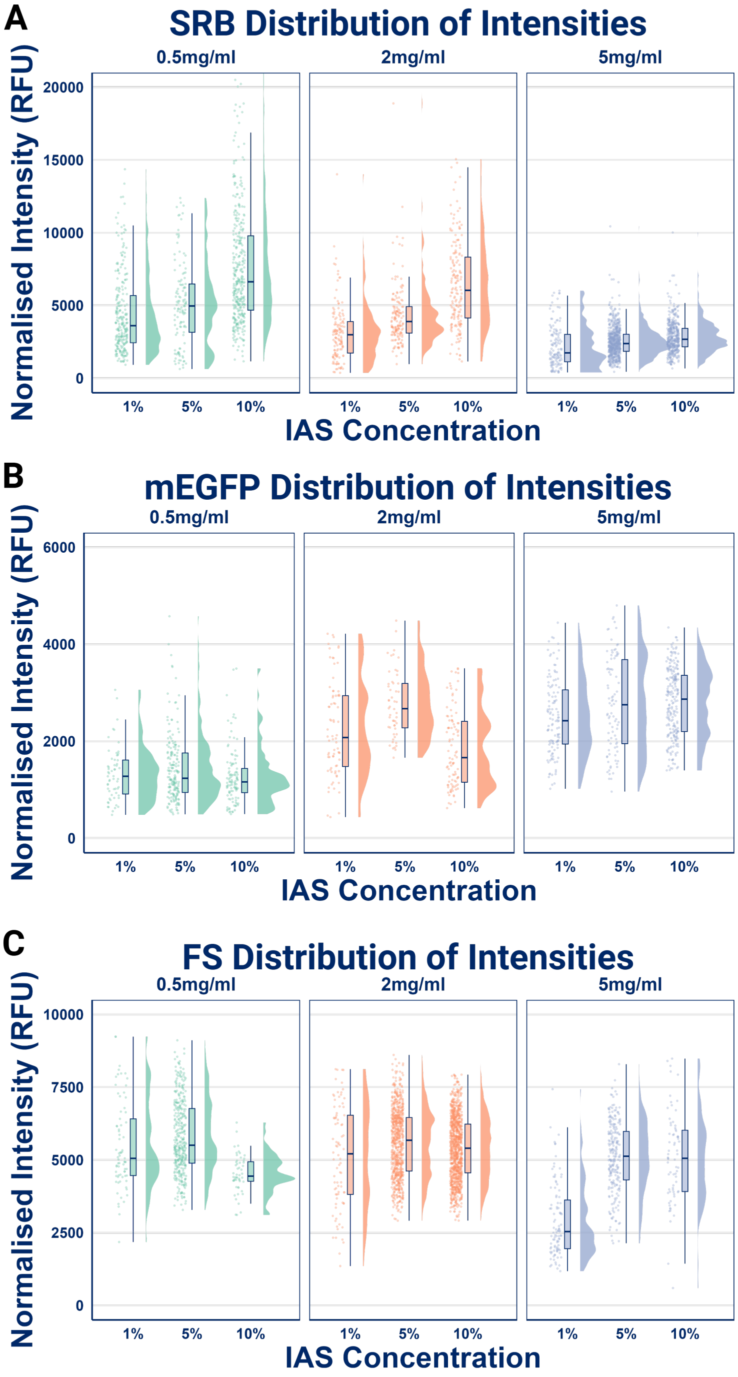

Figure S10: Encapsulation efficiency distributionsA Raincloud plots of SRB encapsulation efficiencies of GUVs containing SRB 580 Da at LiO concentrations of $0.5,\ 2$ *and* $5\ mg/ml$ ***in combination with IAS ratios of*** $1,\ 5$ *and* 10% ***B Raincloud plots of mEGFP encapsulation efficiencies of GUVs containing mEGFP 27 kDa at LiO concentrations of*** $0.5,\ 2$ *and* $5\ mg/ml$ ***in combination with IAS ratios of*** $1,\ 5$ *and* 10% ***C Raincloud plots of FS encapsulation efficiencies of GUVs containing FS 2.6 MDa at LiO concentrations of*** $0.5,\ 2$ *and* $5\ mg/ml$ ***in combination with IAS ratios of*** $1,\ 5$ *and* 10%

Figure S11: Violin plot displaying GUV diameter distribution for each condition tested in figure 4, containing a matrix of LiO concentrations and IAS ratio per fluorescent molecules

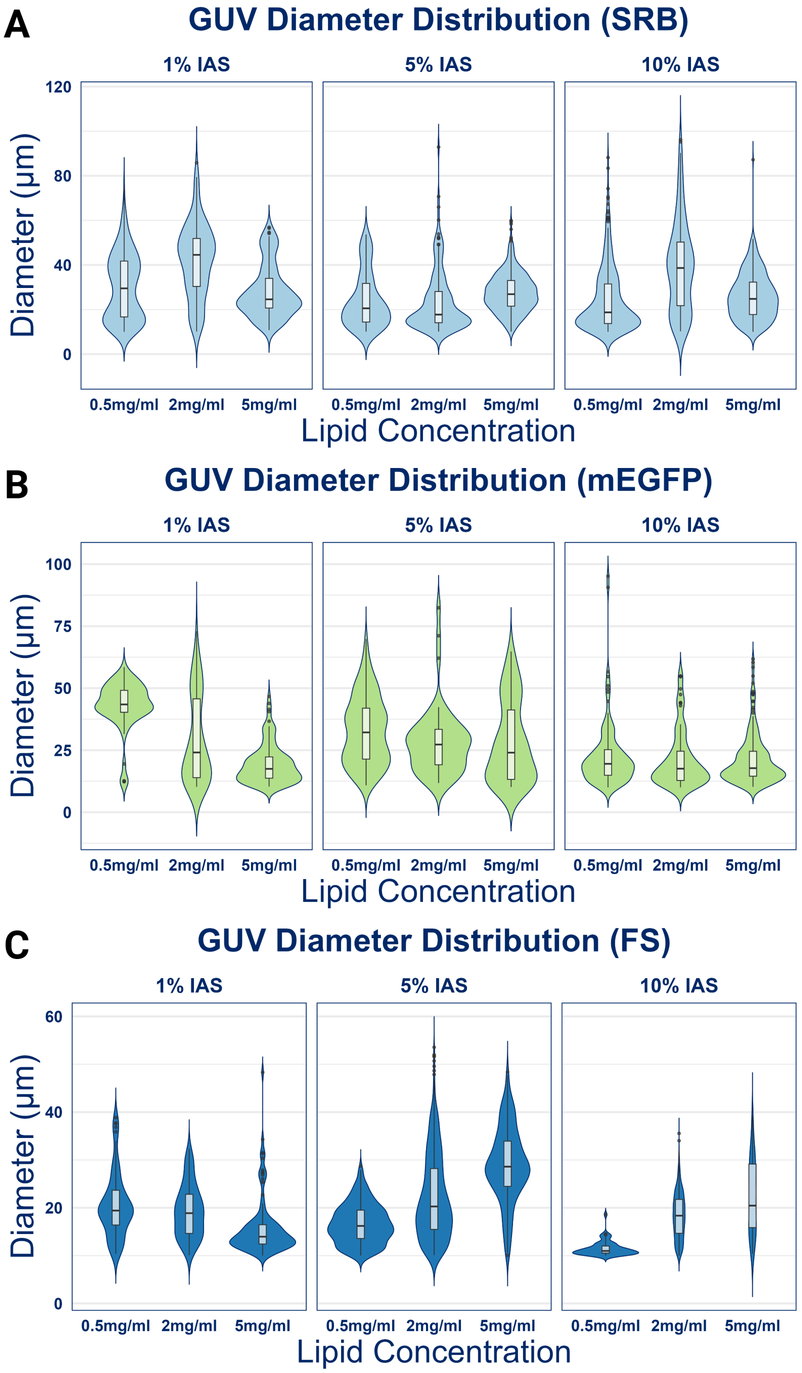

Figure S11: Violin plot of GUV diametersA Violin plot of GUV diameter for vesicles containing SRB 580 Da at LiO concentrations of 0.5, 2 *and* 5 mg/ml ***in combination with IAS ratios of*** 1, 5 *and* 10% ***B Violin plot of GUV diameter for vesicles containing mEGFP 27 kDa at LiO concentrations of*** 0.5, 2 *and* 5 mg/ml ***in combination with IAS ratios of*** 1, 5 *and* 10% ***C Violin plot of GUV diameter for vesicles containing FS 2.6 MDa at LiO concentrations of*** 0.5, 2 *and* 5 mg/ml ***in combination with IAS ratios of*** 1, 5 *and* 10%

Figure S12: Bar plot of an expanded investigation into the effect of PEG and Temperature of encapsulation efficiency

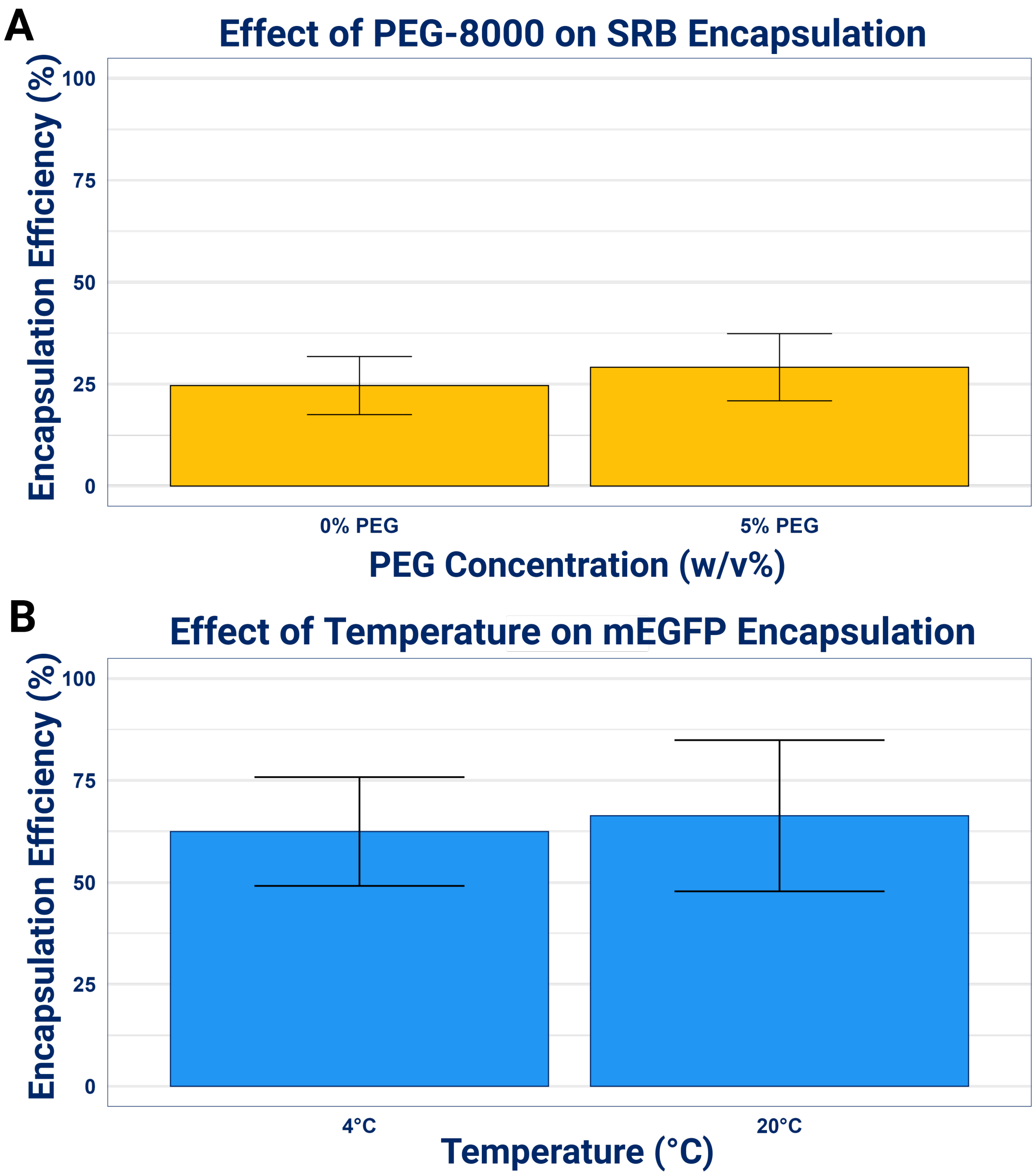

Figure S12: Effect of Temperature and PEG on GUV Encapsulation Efficiency The encapsulation efficiency of GUVs was explored further (A) Bar plot showing the encapsulation efficiencies for two populations of GUVs encapsulating SRB in containing PEG at concentrations of 0 and 5% PEG. Data presented as mean ± standard deviation (SD) from 3 biological repeats. (B) Bar plot showing the encapsulation efficiencies for two populations encapsulation mEGFP within GUVs using a formation temperature of 4 or $20°C$. Data presented as mean ± standard deviation (SD) from 3 biological repeats.

Table S13: Statistical Analysis results for data presented in figure 3. Containing statistical data from a one way ANOVA and post hoc test for each fluorophore

A Lipid effect on SRB Encapsulation
One Way ANOVA Summary

Lipid Conc	N	Mean	SD	Significance Group
0.5mg/ml	121	76.81	41.33	a
2mg/ml	156	69.13	39.76	b
5mg/ml	675	40.18	14.87	c

B Lipid effect on SRB Encapsulation
PostHoc Summary

term	df	sumsq	meansq	statistic	p.value
Lipid Conc	2	208936.52	104468.26	165.50	2.19E-62
Residuals	949	599027.50	631.22	NA	NA

C Lipid effect on mEGFP Encapsulation
One Way ANOVA Summary

Lipid Conc	N	Mean	SD	Significance Group
0.5mg/ml	90	98.45	31.86	a
2mg/ml	52	93.63	23.15	a
5mg/ml	185	50.34	23.61	b

D Lipid effect on mEGFP Encapsulation
PostHoc Summary

term	df	sumsq	meansq	statistic	p.value
Lipid Conc	2	173335.64	86667.82	127.48	1.44E-41
Residuals	324	220266.49	679.83	NA	NA

E Lipid effect on FS Encapsulation
One Way ANOVA Summary

Lipid Conc	N	Mean	SD	Significance Group
0.5mg/ml	359	121.36	20.10	a
2mg/ml	728	115.50	18.00	b
5mg/ml	228	104.31	18.80	c

F Lipid effect on FS Encapsulation
PostHoc Summary

term	df	sumsq	meansq	statistic	p.value
Lipid Conc	2	40725.68	20362.84	58.04	7.01E-25
Residuals	1312	460283.54	350.83	NA	NA

Table S13: Figure 3 Statistical AnalysisA Table containing statistic summary for a one way ANOVA comparing LiO effect on SRB encapsulation efficiency B Table containing statistic summary for a Tukey's post-hoc test comparing the One way ANOVA on LiO effect on SRB encapsulation efficiency C Table containing statistic summary for a one way ANOVA comparing LiO effect on mEGFP encapsulation efficiency D Table containing statistic summary for a Tukey's post-hoc test comparing the One way ANOVA on LiO effect on mEGFP encapsulation efficiency E Table containing statistic summary for a one way ANOVA comparing LiO effect on FS encapsulation efficiency F Table containing statistic summary for a Tukey's post-hoc test comparing the One way ANOVA on LiO effect on FS encapsulation efficiency

Table S14: Statistical Analysis results for data presented in figure 4. Containing statistical data from a two way ANOVA and post hoc test for each fluorophore

A SRB 2 Way ANOVA Summary

term	df	sumsq	meansq	statistic	p.value
Lipid Conc	2	1623527.48	811763.74	620.66	1.49E-218
IAS	2	494298.16	247149.08	188.96	5.15E-77
Lipid Conc:IAS	4	191933.58	47983.40	36.69	8.01E-30
Residuals	2435	3184763.05	1307.91	NA	NA

B SRB PostHoc Summary

Lipid Conc	IAS	N	Mean	SD	Significance Group
0.5 mg/ml	10%	356	126.19	63.95	a
2 mg/ml	10%	169	103.01	49.44	b
0.5 mg/ml	5%	121	76.81	41.33	c
0.5 mg/ml	1%	250	74.19	40.13	c
2 mg/ml	5%	156	69.13	39.76	c
2 mg/ml	1%	135	54.36	33.45	d
5 mg/ml	10%	444	46.78	15.38	de
5 mg/ml	5%	675	40.18	14.87	e
5 mg/ml	1%	138	36.04	22.22	e

C mEGFP 2 Way ANOVA Summary

term	df	sumsq	meansq	statistic	p.value
Lipid Conc	2	452596.60	226298.30	381.82	5.09E-124
IAS	2	11586.03	5793.01	9.77	6.25E-05
Lipid Conc:IAS	4	26027.30	6506.83	10.98	1.02E-08
Residuals	1003	594454.19	592.68	NA	NA

D mEGFP PostHoc Summary

Lipid Conc	IAS	N	Mean	SD	Significance Group
5 mg/ml	10%	225	99.11	20.84	a
5 mg/ml	5%	90	98.45	31.86	ab
2 mg/ml	5%	52	93.63	23.15	abc
5 mg/ml	1%	119	88.00	25.87	bc
2 mg/ml	1%	76	80.98	32.84	c
2 mg/ml	10%	96	67.14	24.47	d
0.5 mg/ml	5%	185	50.34	23.61	e
0.5 mg/ml	1%	59	46.70	18.26	e
0.5 mg/ml	10%	110	44.35	19.35	e

E FS 2 Way ANOVA Summary

term	df	sumsq	meansq	statistic	p.value
Lipid Conc	2	150878.99	75439.49	178.51	1.48E-73
IAS	2	85121.42	42560.71	100.71	6.47E-43
Lipid Conc:IAS	4	111978.39	27994.60	66.24	1.55E-53
Residuals	2710	1145249.88	422.60	NA	NA

F FS PostHoc Summary

Lipid Conc	IAS	N	Mean	SD	Significance Group
0.5mg/ml	5%	359	121.36	20.10	a
0.5mg/ml	1%	73	120.49	25.51	ab
2mg/ml	5%	728	115.50	18.00	b
2mg/ml	10%	1025	110.38	20.65	c
2mg/ml	1%	84	106.02	30.20	cd
5mg/ml	5%	228	104.31	18.80	d
5mg/ml	10%	71	102.86	29.32	cd
0.5mg/ml	10%	52	90.57	11.13	e
5mg/ml	1%	99	60.76	24.63	f

Table S14: Figure 4 Statistical AnalysisA Table containing statistic summary for a two way ANOVA comparing LiO effect on SRB encapsulation efficiency B Table containing statistic summary for a Tukey's post-hoc test comparing the two way ANOVA on LiO effect on SRB encapsulation efficiency C Table containing statistic summary for a two way ANOVA comparing LiO effect on mEGFP encapsulation efficiency D Table containing statistic summary for a Tukey's post-hoc test comparing the two way ANOVA on LiO effect on mEGFP encapsulation efficiency E Table containing statistic summary for a two way ANOVA comparing LiO effect on FS encapsulation efficiency F Table containing statistic summary for a Tukey's post-hoc test comparing the two way ANOVA on LiO effect on FS encapsulation efficiency

Table S15: Statistical Analysis results for data presented in figure 5A. Containing statistical data from a One way ANOVA and post hoc test for the effect of PEG on mEGFP encapsulation efficiency

A PEG Concentration one Way ANOVA Summary

term	df	sumsq	meansq	statistic	p.value
PEG_Conc	3	318016.83	106005.61	373.45	3.88E-210
Residuals	3463	982981.61	283.85	NA	NA

B PEG Concentration PostHoc Summary

PEG Conc	N	Mean	SD	Significance Group
5%	177	107.27	19.52	a
4%	1182	70.18	14.89	b
3%	1653	66.63	15.28	c
0%	455	58.73	24.32	d

Table S15: Figure 5A Statistical AnalysisA Table containing statistic summary for a one way ANOVA comparing the effect of PEG concentration mEGFP encapsulation efficiency B Table containing statistic summary for a Tukey's post-hoc test comparing the one way ANOVA on the effect of PEG concentration mEGFP encapsulation efficiency

Table S16: Statistical Analysis results for data presented in figure 5B. Containing statistical data from a two tail independent T-Test for the effect of temperature on SRB encapsulation efficiency

Variable Compared	Group 1	Group 2	t-statistic (t)	Degrees of Freedom (df)	Sample Size (n1,n2)	p-value (p)
Encapsulation Percent	4	20	-19.9	2825	1406, 1444	< 0.001

Table S16: Figure 5B Statistical Analysis A table containing statistical data from a two tail independent T-Test for the effect of temperature on SRB encapsulation efficiency

Figure S17: Size vs Encapsulation efficiency analysis for GUV populations figures 3 and 4. a multi-panel grid of scatter plots with linear regression lines, designed to show the relationship between GUV physical characteristics and their encapsulation performance across different molecule types and formation conditions.

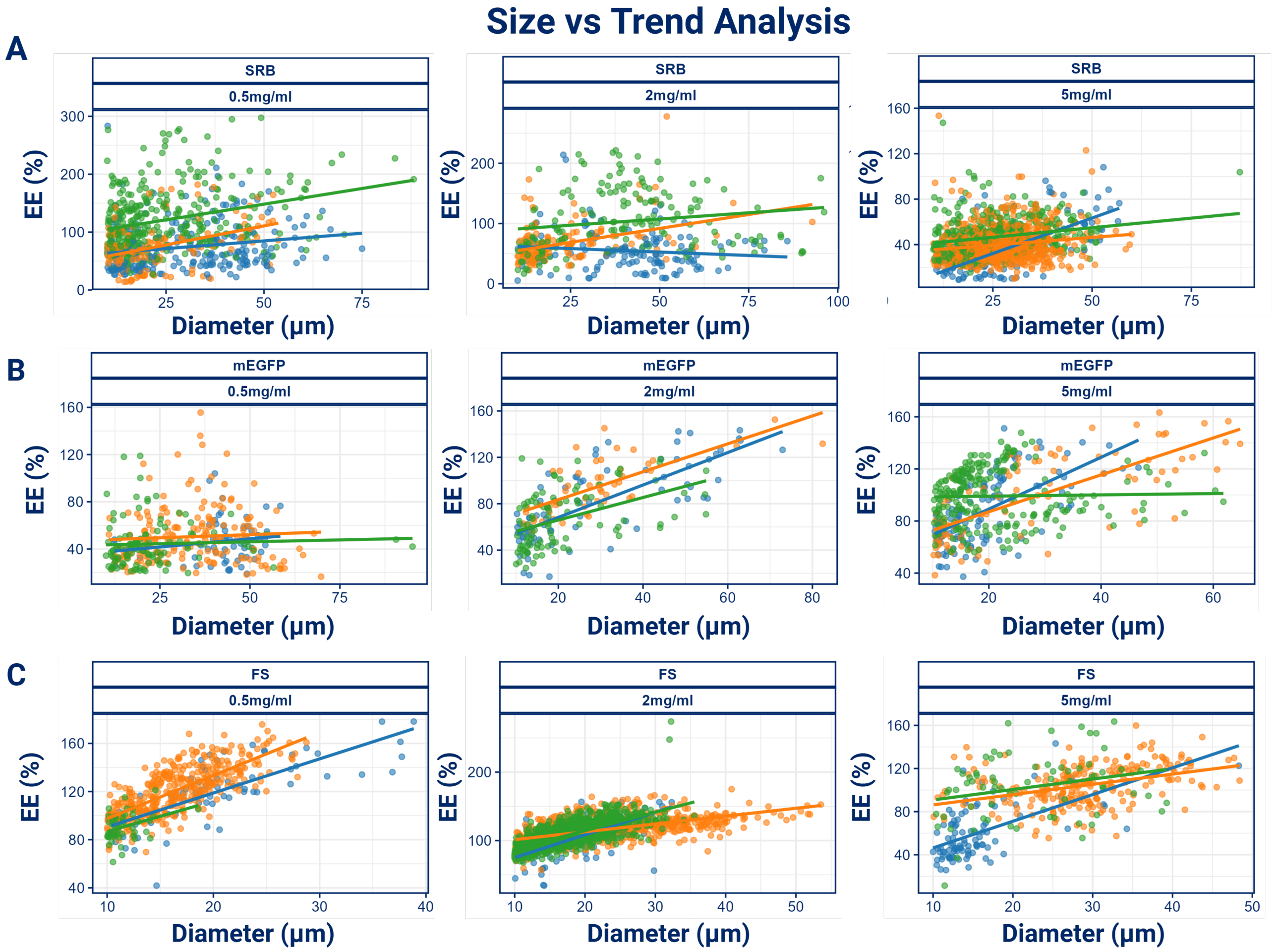

Figure S17: Scatter plots with linear regression showing Size vs Encapsulation efficiency analysis for GUV populationsA Scatter plots of GUV diameter vs Encapsulation efficiency for vesicles containing SRB 580 Da at LiO concentrations of $0.5, 2$ *and* 5 *mg/ml* ***in combination with IAS ratios of*** $1, 5$ *and* 10% ***B Scatter plots of GUV diameter vs Encapsulation efficiency for vesicles containing mEGFP 27 kDa at LiO concentrations of*** $0.5, 2$ *and* 5 *mg/ml* ***in combination with IAS ratios of*** $1, 5$ *and* 10% ***C Scatter plots of GUV diameter vs Encapsulation efficiency for vesicles containing FS 2.6 MDa at LiO concentrations of*** $0.5, 2$ *and* 5 *mg/ml* ***in combination with IAS ratios of*** $1, 5$ *and* 10%

Text S18: GFP Production Protocol pRSET his-eGFP was a gift from Jeanne Stachowiak Addgene plasmid # 113551 http://n2t.net/addgene:113551 ; RRID: Addgene_113551.

Plasmids were transformed into competent E. coli (BL21) cells and single colonies were selected to inoculate a 50 mL culture of LB (100 µg/mL ampicillin) at $37°C$, 250 RPM. Protein production was induced with the addition of IPTG to a final concentration of 1 mM and incubated for 16 h at $30°C$, 250 RPM. Bacteria were harvested by centrifugation at 4000 RPM and the pellets stored at $-80°C$.

The cell pellet was resuspended in B-Per complete bacterial protein extraction reagent (ThermoFisher) and centrifuged at 4000 RPM for 15 min. Purification was performed by HisPur superflow Ni-NTA agarose beads under conditions described by the manufacturer (thermoscientific) using Pierce™ Disposable Columns, 5 mL. Purified protein was eluted with 250 mM imidazole, the elution buffer was exchanged to PBS and concentrated with Amicon Ultra Centrifugal filters (Millipore Ultracel-10 K) with a 10 kDa cut off. Concentrated samples in PBS were stored at $4°C$.Samples were concentrated to 66 µM using Beer-lambert law:

$$A = \varepsilon cl$$

Where A is the absorbance at the excitation maximum (510 nm), e is the extinction coefficient in (M-1 cm-1), l is the pathlength in cm and C is the Molar concentration.
